# Sephin1 suppresses ER stress-induced cell death by inhibiting the formation of PP2A holoenzyme

**DOI:** 10.1038/s41419-025-07450-1

**Published:** 2025-02-19

**Authors:** Satoshi Gojo, Daisuke Kami, Arata Sano, Fumiya Teruyama, Takehiro Ogata, Satoaki Matoba

**Affiliations:** 1 Department of Regenerative Medicine, Graduate School of Medicine, Kyoto Prefectural University of Medicine, Kyoto, Japan; 2 Department of Cardiovascular Medicine, Graduate School of Medicine, Kyoto Prefectural University of Medicine, Kyoto, Japan; 3 Tokyo New Drug Research Laboratories, Kowa Company Ltd., Tokyo, Japan; 4 Department of Pathology and Cell Regulation, Graduate School of Medicine, Kyoto Prefectural University of Medicine, Kyoto, Japan

**Keywords:** Drug development, Acute kidney injury

## Abstract

Sephin1 was discovered as a protein phosphatase inhibitor, and its efficacy against neurodegenerative diseases has been confirmed. There are conflicting reports on whether inhibition of eIF2α dephosphorylation by PP1 holoenzyme with the protein phosphatase 1 regulatory subunit 15 A is the mechanism of action of Sephin1. In the present study, we found that Sephin1 significantly suppressed renal tubular cell death in an animal model of ER stress administered with tunicamycin. CHOP, which plays a central role in the ER stress-induced cell death pathway, requires nuclear translocation to act as a transcription factor to increase the expression of cell death-related genes. Sephin1 markedly suppressed this nuclear translocation of CHOP. To elucidate the molecular mechanism underlying the cell death suppressive effect of Sephin1, we used human renal tubular epithelial cells under ER stress with tunicamycin. Sephin1 reduced intracellular CHOP levels by promoting CHOP phosphorylation at Ser30, which led to protein degradation in UPS. Phosphorylated CHOP is generated by Thr172-phosphorylated activated AMPK, and Sephin1 increased phosphorylated AMPK. Phosphorylated AMPK is inactivated by PP2A through dephosphorylation of its Thr172, and Sephin1 inhibits the formation of the PP2A holoenzyme with the PP2A subunit B isoform delta. These results indicate that inhibition of PP2A holoenzyme formation is the molecular target of Sephin1 in this experimental system.

## Introduction

Various biological processes, as well as intracellular signal transduction, occur by the addition and removal of phosphate groups. Substances that inhibit the function of kinases, which add phosphate groups, have been developed as targets for drug discovery, and many have been used in clinical practice. On the other hand, substances that inhibit the function of phosphatases, which remove phosphate groups, have been slow to develop as drug discovery targets [1]. Sephin1 was reported in 2015 to intervene in the eIF2α response system, which is located downstream of PERK in the unfolding protein response (UPR) and where the integrated stress response (ISR) converges, to demonstrate pathomechanical improvement in neurodegenerative diseases [2]. Phosphorylated eIF2α is a substrate for dephosphorylation by a holoenzyme comprising a protein phosphatase 1 (PP1) catalytic subunit and a regulatory subunit. This reaction may inhibit excess UPR but may also inhibit the necessary UPR in some cases [3]. The study showing the effect of Sephin1 on Charcot-Marie-Tooth 1B and amyotrophic lateral sclerosis suggests that prevention of inappropriate UPR resolution and maintenance of an appropriate UPR are necessary for disease improvement in an environment where the underlying pathological cause has not been eliminated. The point of action of Sephin1 was shown to inhibit holoenzyme formation by binding to the PP1 catalytic subunit and the protein phosphatase 1 regulatory subunit 15 A (PPP1R15A, known as Growth arrest and DNA damage-inducible protein: GADD34), which in turn inhibited the dephosphorylation of phosphorylated eIF2α [2]. The dephosphorylation of phosphorylated eIF2α was shown to be mediated by the GADD34 scaffold, which bound the PP1 catalytic subunit via RVxF and Φ/Φ motifs and eIF2α via PEST repeats, resulting in dephosphorylation [4]. In contrast, it has been reported that Sephin1 does not inhibit the association between the PP1 catalytic subunit and GADD34 and consequently the dephosphorylation of phosphorylated eIF2α by the holoenzyme [5]. In addition to holoenzyme formation inhibition, G-actin is required for eIF2α dephosphorylation, and the holoenzyme comprising of these three proteins is not inhibited by Sephin1 [6]. It has also been reported that the protection of neurons from excitotoxicity by Sephin1 is an event independent of the ISR [7]. In response to these conflicting reports, it has been considered necessary to investigate an alternative mode of action for Sephin1 that clearly improves its pathophysiology.

In order to elucidate mechanisms other than the initially reported point of action of Sephin1, we decided to test a model in which tunicamycin, which induces ER stress, is administered to mice. In this model, renal damage is reported to be frontline and the pathology is tubular epithelial cell death [8, 9]; if Sephin1 is able to inhibit this cell death, we thought we could validate the mode of action in an in vitro system, given the availability of a tubular epithelial cell line.

## Results

### Sephin1 rescues tunicamycin-induced ER stress

**Fig. 1:**
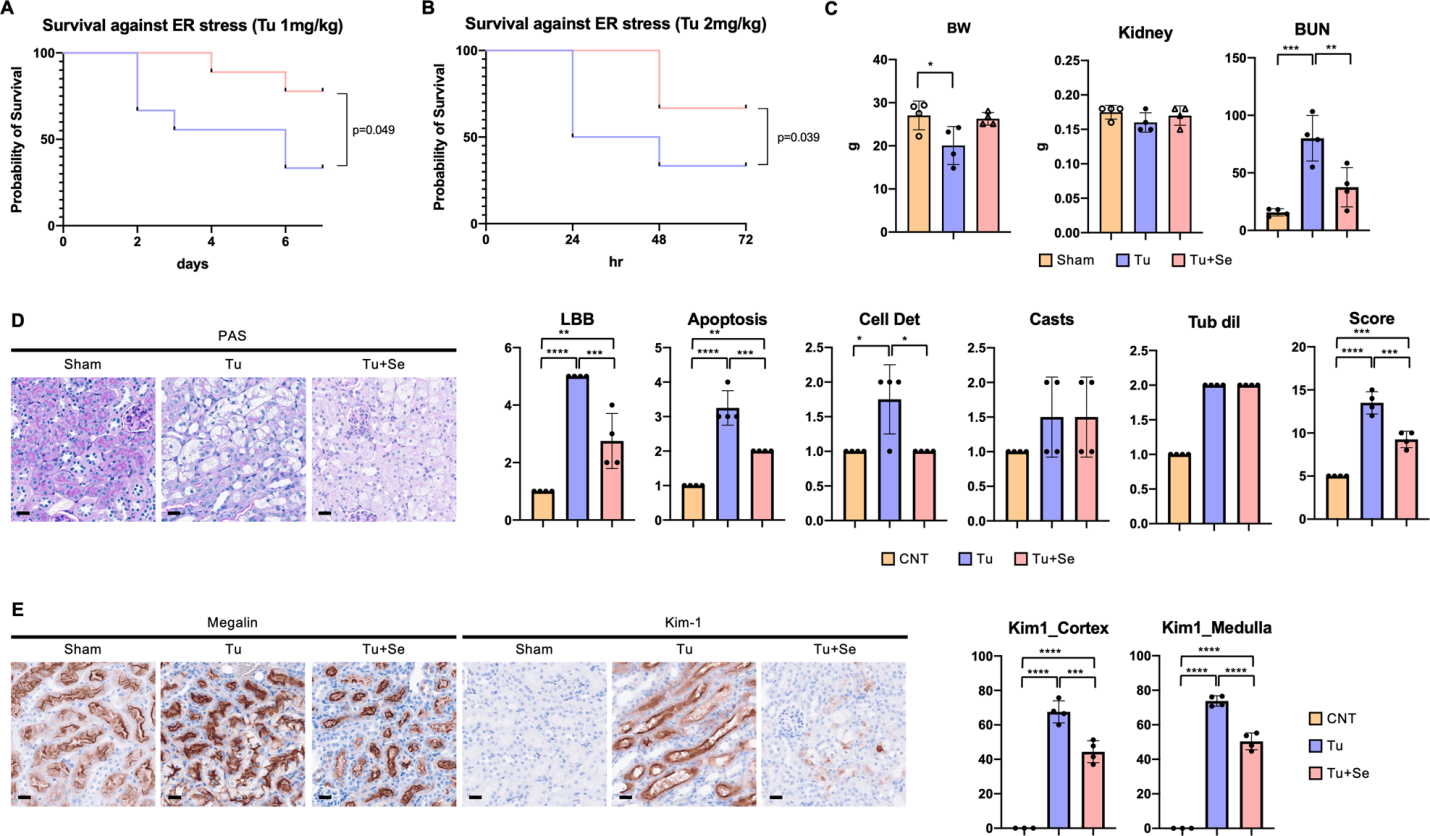
Sephin1 rescues tunicamycin-induced ER stress. **A**, **B** Kaplan–Meier survival curves of mice receiving tunicamycin intraperitoneal injections at the following concentrations without or with Sephin1; **A** 1 mg/kg, *n* = 9, **B** 2 mg/kg, *n* = 12. **C** Body, kidney weight, and BUN were measured on Day 3 (*n* = 4). **D** Representative images of PAS staining of the kidney of mice on Day 3 and quantification of kidney injuries (*n* = 4). **E** Representative images of Megalin staining and Kim-1 staining of the kidney of mice on Day 3 and the Kim-1 positivity as damaged tubular epithelium (*n* = 4). The error bars indicate the SDs. The number of asterisks can be obtained by indicating a range of *p* values as follows; * <0.05, ** <0.01, *** <0.001, **** <0.0001.

Many studies have been conducted on ER stress caused by tunicamycin-inhibiting glycosylation in the ER, which is related to various pathology [10]. The kidney has been reported to be affected by direct administration of tunicamycin to animals [11–14]. The first step was to test the protective effect of Sephin1 against organ damage caused by the intraperitoneal administration of tunicamycin in mice. The administration protocol consisted of a single intraperitoneal dose of tunicamycin (defined as Day 0) followed by a once-daily intraperitoneal dose of 4 mg/kg [15] of Sephin1 for 3 consecutive days to verify survival (Fig. S1A). When tunicamycin was administered at a dose of 1 mg/kg [12], half of the mice in the vehicle group died on Day 3, whereas all mice in the Sephin1 group treated with 1 mg/kg survived on Day 3. After observation until Day 7, Kaplan–Meier survival curve was drawn and the log-rank (Mantel-Cox) test was performed, which confirmed significantly better survival in the Sephin1-treated group compared with the sham group (Fig. 1A). In the experiment with 2 mg/kg tunicamycin [11], a Kaplan–Meier survival curve was drawn for an observation period of up to 72 h, and the difference was even more evident than that with 1 mg/kg tunicamycin (Fig. 1B). At Day 3, the body weight of mice in the 1 mg/kg tunicamycin protocol was significantly lower than in the sham group, and the mice recovered with Sephin1 treatment, although there is no significant difference (*p* = 0.062) (Fig. 1C). The weights of the kidney relative to body weight did not significantly change (Fig. 1C). As surrogate markers of renal function, BUN was measured in blood samples. BUN showed significantly impaired organ function in the tunicamycin-treated group and significantly improved in the Sephin1-treated group (Fig. 1D).

**Fig. 2:**
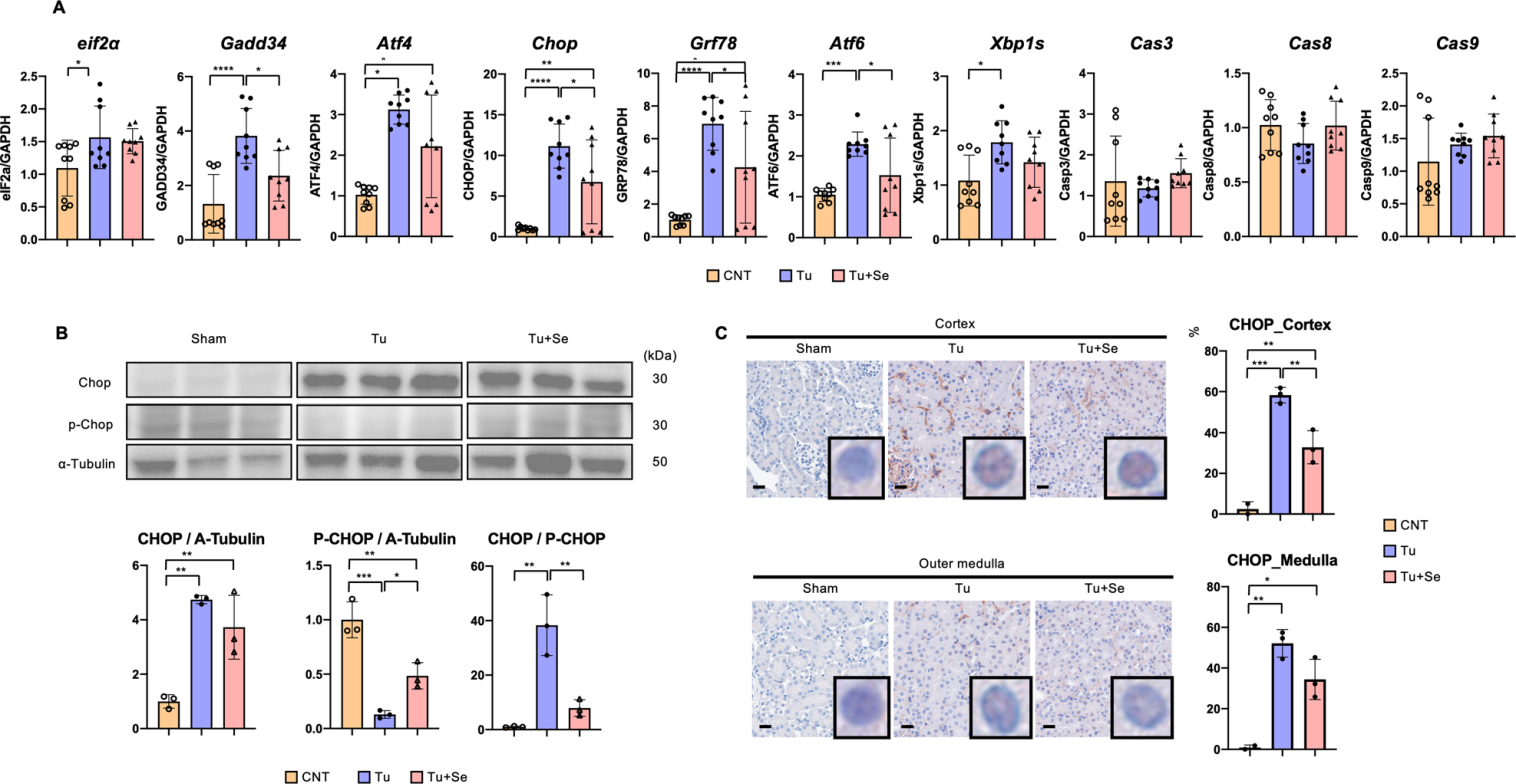
Expression of CHOP induced by ER stress is suppressed by Sephin1. **A** The mRNA expression of murine kidney on Day 3 (*n* = 9). The error bars indicate the SDs. **B** Western blotting analysis of CHOP in murine kidneys on Day 3. The graph shows the expression ratio of each protein corrected by α-Tubulin protein expression level. **C** Representative images of CHOP staining of murine kidney on Day 3 and the CHOP-positive rate in the nucleus in either the cortex or medulla (*n* = 3). All bars in the images are 100 μm. The number of asterisks can be obtained by indicating a range of *p* values as follows; * <0.05, ** < 0.01, *** <0.001, **** <0.0001.

In the animal study involving 1 mg/kg tunicamycin, tunicamycin-induced tubular damage was significant on Day 3, and treatment with Sephin1 significantly ameliorated the pathological changes, based on PAS staining (Fig. 1D, Fig. S1B–D). To quantify these pathological changes, we quantified the following parameters [16]; loss of brush border (LBB), loss of tubular cells detachment from the basement membrane (Cell Det), apoptosis/necrosis, presence of vitreous material (Casts) in the tubular lumen, and tubular dilatation (Tub Dil) (Fig. 1D). The tunicamycin group showed a significant increase in the loss of brush border, tubular cell shedding from the basement membrane, and apoptosis, which were significantly decreased by Sephin1 treatment. On the other hand, the presence of vitreous substances and tubular dilation were found to be elevated in the tunicamycin group, but were not significantly reduced by Sephin1 treatment. The total score, which is representative of the degree of tubular damage calculated by adding these five parameters, was significantly impaired with tunicamycin and significantly reduced with Sephin1 (Fig. 1D). Next, the damaged tubules were identified by immunostaining with Megalin expressing in the entire tubular cells and Kim-1 expressing in the damaged tubular cells [17]. Tunicamycin caused impaired tubular cells to reach 70% of the total number of cells, whereas Sephin1 treatment reduced this impairment to approximately 50% (Fig. 1E, Fig. 2A–C). Sephin1 rescued the damage with a significant difference from the tunicamycin group in either the medullary or cortical regions. The protective effect of Sephin1 in the kidney was mainly due to the inhibition of tubular epithelial cell death, and other constituent cell groups, such as the vascular system and stroma, were not significantly altered.

### Identification of molecular targets of Sephin1

Gene expression in the kidney on Day 3 was examined for UPR-related, mitochondria-related, and cell death-related genes (Fig. 2A, Fig. S3). The expression of genes related to ER stress was enhanced by tunicamycin treatment, albeit to varying degrees. In particular, *Chop* was the most significantly upregulated, while *eIF2α* was the least upregulated of the genes tested. Sephin1 treatment decreased the expression of *Chop, Gadd34, Grp78*, and *Atf6*, with statistically significant differences. The gene expression of *Atf6* was suppressed by Sephin1, whereas the gene expression of *Xbp1s*, which is strongly affected by ATF6 [18], was not significantly reduced by Sephin1. This suggests that the point of action of Sephin1 in this experimental system is the PERK pathway rather than IRE1a or ATF6 in the UPR. On the other hand, neither tunicamycin nor Sephin1 treatment significantly altered *Caspase3* as an apoptosis executor, *Caspase9* in the mitochondrial pathway, or *Caspase8* in the non-mitochondrial pathway in terms of gene expression. Because Sephin1 averted cell death on pathological examination, the cell death-related mRNA expression results suggest that the intervention of Sephin1 could affect the protein expression for cell death-related factors. *Chop* is regulated by post-translational modification of its activity to act as a transcription factor in the nucleus [19] and is a direct regulator of cell death [20]. Next, we decided to test the hypothesis that Sephin1 may be involved in the regulation of cell death via intervention in the nuclear translocation of CHOP.

To examine that the mode of action of Sephin1 against ER stress in this study was via the pancreatic ER kinase (PERK) pathway, the phosphorylation status of eIF2a was quantified by western blotting (WB) (Fig. S4). In this animal model, Eif2α was significantly increased by tunicamycin treatment, whereas Sephin1 had no significant effect on its protein levels. As for phosphorylated Eif2α, little change was induced in the tunicamycin-treated group. However, the results that phosphorylated eIF2α was lower in the tunicamycin- and Sephin1-treated groups than in the sham group suggests that at this time point, the cell death phenotype has already passed its climax and ER stress may be in an off phase in the cells. Cell death induced by ER stress is closely related to CHOP, and the translocation of CHOP to the nucleus transcriptionally induces cell death-related factors [20]. The CHOP protein was also significantly increased by tunicamycin treatment and decreased in the Sephi1-treated group, although not significantly (Fig. 2B). Phosphorylated CHOP, which was evaluated by the antibody recognizing the phosphorylation of CHOP Ser30, significantly increased in the Sephin1-treated group, and the CHOP/phosphorylated CHOP ratio significantly decreased, compared with that in the tunicamycin-treated group. These results indicate that the increased CHOP in ER stress decreased by its decay in the UPS [20].

Next, we examined the nuclear translocation of CHOP via immunostaining because CHOP migrates to the nucleus and acts as a transcriptional factor that promotes the expression of cell death-related genes. Mice were treated with only one dose of 2 mg/kg tunicamycin without or with 4 mg/kg Sephin1, and kidneys were harvested 12 h later for pathological examination because the nuclear transfer of transcriptional factors occurs in a kinetic manner, such as within minutes to hours in response to a stimulus [21, 22]. The positive rate of CHOP in the nucleus was calculated for the cortex and medulla (Fig. 2C, Fig. S5A–C). In the sham group, CHOP is rarely found in the nucleus, but both in the cortex and medulla can be recognized in approximately 60% of tubular cells by ER stress. In the Sephin1 treatment group, its percentage was decreased in both areas, with a significant decrease in the cortex. These results suggest that phosphorylation of CHOP Ser30 causes protein decay in the cytoplasm, reducing its total protein content and lowering the amount of CHOP that migrates to the nucleus.

### In vitro analysis of CHOP regulation by Sephin1

**Fig. 3:**
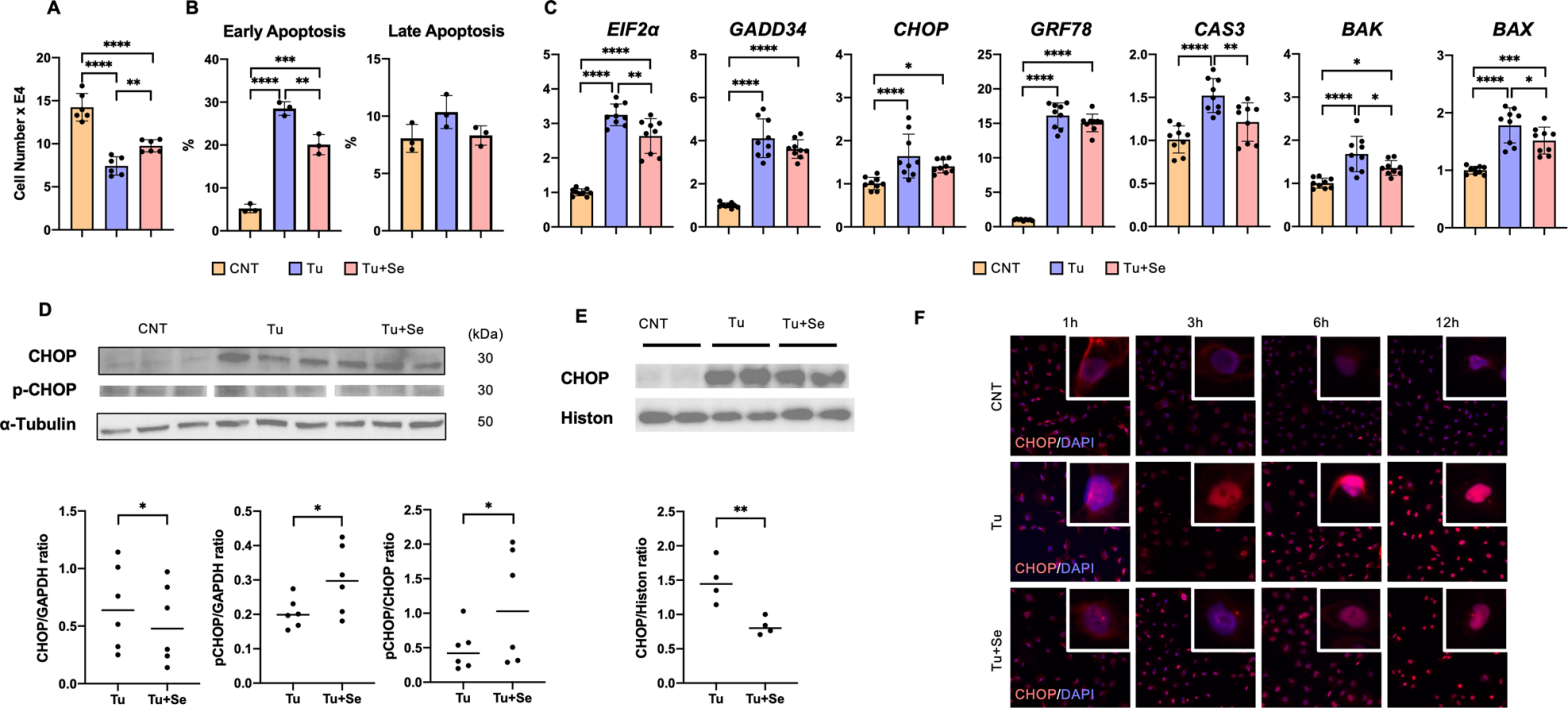
In vitro analysis of CHOP regulation by Sephin1. **A** Number of HK-2 cells exposed to 20 μg/ml tunicamycin without or with 2 μM Sephin1 for 24 h (*n* = 3). **B** Apoptosis analysis using PI and annexin V of HK-2 cells at 24 h following the administration of 1 mg/ml tunicamycin without or with 1 μM Sephin1 by FACS (*n* = 3). **C** The mRNA expression of HK-2 cells at 24 h following the administration of 20 μg/ml tunicamycin without or with 1 μM Sephin1 (*n* = 3). **D** Western blotting analysis of CHOP and eIF2α of HK-2 cells at 24 h following the administration of 20 μg/ml tunicamycin without or with 1 μM Sephin1. The graph shows the expression ratio of each protein corrected by α-Tubulin protein expression level (*n* = 3). **E** Western blotting analysis of CHOP in the nuclear fraction of HK-2 cells at 24 h following the administration of 20 μg/ml tunicamycin without or with 1 μM Sephin1 (*n* = 3). **F** Immunocytochemistry using anti-CHOP antibody to HK-2 cells over time. The error bars indicate the SDs. The error bars indicate the SDs. The number of asterisks can be obtained by indicating a range of *p* values as follows; * <0.05, ** <0.01, *** <0.001, **** <0.0001.

The results of animal studies in the ER stress model with tunicamycin showed that renal tubular epithelial cell death is key to pathogenesis and prognosis, and that Sephin1 inhibits its cell death and significantly suppresses the nuclear migration of CHOP, which is essential for cell death induction. The molecular mechanism of nuclear migration of CHOP was investigated in an in vitro cell culture model. We proceeded to examine it in an ER stress model using a human tubular epithelial cell, HK-2. Tunicamycin at 20 μg/ml significantly suppressed cell numbers of HK-2 at 24 h, and 2 μM Sephin1 treatment significantly restored cell numbers (Fig. 3A). To verify the stage of cell death at which Sephin1 was rescued, we performed staining with PI and annexin V and analyzed the cells by FACS (Fig. 3B, Fig. S6A). Exposure to 20 μg/ml tunicamycin for 24 h increased both the percentage of annexin V-positive and PI-negative cells, which is a phenotype of early apoptosis, and the percentage of annexin V and PI double positive cells, which indicates late apoptosis, but the difference was not significant. We also created an ER stress model by adding Brefedlin, which indirectly induces ER stress by inhibiting Golgi transport, and thapsigargin, which impairs ER Ca storage, to HK-2 and confirmed the effect of Sephin1 on them (Fig. S6B). A cytoprotective effect was observed for Brefedlin, like the effect on tunicamycin. For thapsigargin, on the other hand, the effect was examined at two time points, 3 h and 24 h, the same as in the model where tunicamycin was used. However, at both time points, the effect of Sephin1 was not significantly cytoprotective, although there was a trend towards recovery in cell numbers. This result suggests that Sephin1 may not have a sufficient effect on Ca-induced ER stress or, given the mechanism of action of the two compounds, on acute stress.

Next, we examined whether Sephin1 affects the role of CHOP as a transcription factor in the HK-2 ER stress model via qPCR for transcripts of cell death-related genes downstream of CHOP, mitochondria-related genes, and UPR pathway-related genes (Fig. 3C, Fig. S6C). All three pathways of the UPR showed significant increases in mRNA expression after 24 h of tunicamycin exposure, but only *eIF2α* was significantly downregulated by Sephin1. Sephin1 did not cause significant changes in ATF6 and significantly reduced Xbp1s and Xbp1u (Fig. S6C). In addition, GRP94, which binds to PERK in the ER lumen and contributes to its inactivation, is reduced by Sephin1. The inconsistency with qPCR results in animal studies may be due to the fluctuation of the UPR. *CHOP* showed no significant change in response to Sephin1, suggesting that Sephin1 acts through post-translational modification rather than transcriptional regulation. In contrast to these changes, the expression of cell death-related genes was significantly altered: caspase 3, an executor of apoptosis, and Bak and Bax, intrinsic apoptosis pathways, were significantly elevated by tunicamycin exposure and significantly decreased by Sephin1 treatment. This indicates the effect of Sephin1 on the transcriptional regulation of cell death-related genes by CHOP.

Protein expression was investigated in the same system as HK-2 to examine whether Sephin1 is involved in the post-translational modification of CHOP. CHOP, which was increased by tunicamycin, was significantly decreased by Sephin1 treatment (Fig. 3D, Fig. S6B). CHOP phosphorylated at Ser30 was unchanged by tunicamycin exposure and significantly increased by Sephin1 treatment. The decrease in CHOP at the protein level could be due to the decay of CHOP, as there was no decrease in *CHOP* transcripts (Fig. 3C) and an increase in phosphorylated CHOP at Ser30, which that is degraded in UPS. Even the ratio of phosphorylated CHOP to CHOP was significantly increased in the tunicamycin-exposed group. The total amount of CHOP protein in the cytoplasm is reduced by its decay, resulting in reduced nuclear translocation of CHOP, as demonstrated by quantifying CHOP in the nuclear fraction (Fig. 3E). Because the kinetics of this nuclear translocation is assumed to be very rapid, we investigated CHOP nuclear translocation by immunocytochemically staining intracellular CHOP over time (Fig. 3F, Fig. S7). The nuclear migration started 3 h after exposure to tunicamycin, and strong coloration was observed even at 12 h, the last time the study was conducted. In contrast, the presence of CHOP was barely detectable at 3 h after Sephin1 treatment but was slightly visible at 6 h and slightly increased at 12 h. These results indicate that Sephin1 is a reliable regulator of CHOP. Whether cell death in this system is uniquely dependent on CHOP regulation and whether Sephin1 is involved in its regulation is the next question.

### Effects of mitochondrial functional by Sephin1

**Fig. 4:**
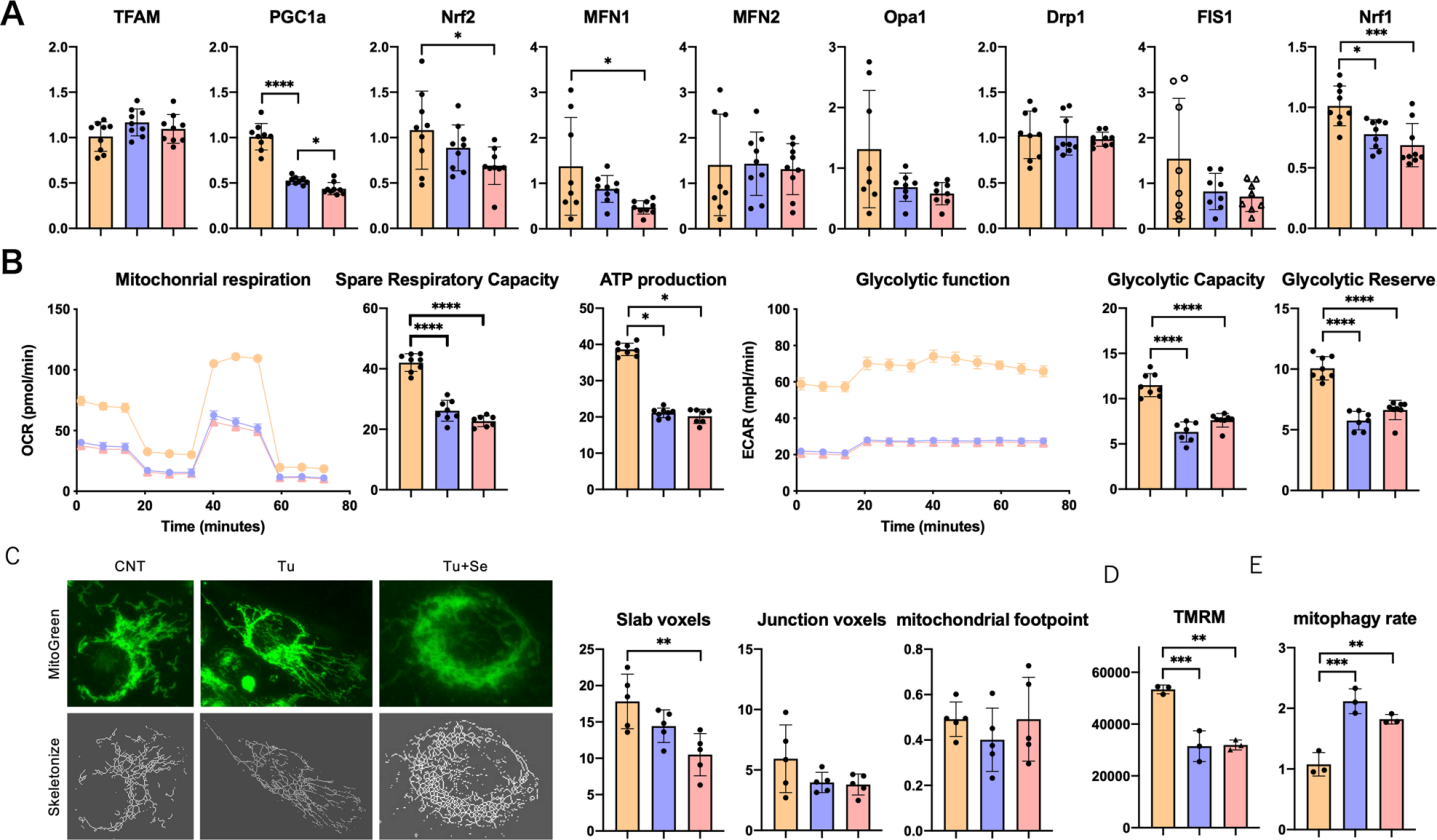
Effects of mitochondrial functional by Sephin1. **A** The mitochondria-related mRNA expression of HK-2 cells at 24 h following the administration of 20 μg/ml tunicamycin without or with 1 μM Sephin1 (*n* = 3). **B** Oxygen consumption rates and glycolysis analysis using Seahorse in HK-2 cells under ER stress model (*N* = 7). **C** Mitochondrial network analysis in HK-2 cells following TMRM staining (*N* = 4). **D** Mitochondrial membrane potential in HK-2 cells by FACS analysis of TMRM staining (*N* = 3). **E** Mitophagy analysis using mKeima expressing HK-2 cells by FACS analysis (*N* = 3). The number of asterisks can be obtained by indicating a range of *p* values as follows; * <0.05, ** <0.01, *** <0.001, **** <0.0001.

ER stress sends a stress signal to mitochondria and modifies mitochondrial function [23], and mitochondrial dysfunction sends a stress signal to the ER signaling interactions have important implications for biological processes, especially cell death [24]. About the mechanism of action of Sephin1, we examined the effect of Sephin1 on mitochondrial function under ER stress. Transcripts involved in mitochondria under ER stress in HK-2 cells were examined (Fig. 4A). Sephin1 did not increase the transcripts of PGC1α or TFAM, which are involved in mitochondria biogenesis. With respect to dynamics, Sephin1 further significantly reduced expression of MFN1. The results that MFN2 and OPA1, which is involved in fusion, and Drp1 and Fis1, which are involved in fission, are not altered, suggest that Sephin1 might not influence mitochondrial dynamics (Fig. 4A). Next, we examined the changes in mitochondrial respiratory capacity and glycolytic activity under ER stress (Fig. 4B). We found that both metabolisms were severely restricted by ER stress, but Sephin1 had no significant effect on these metabolic alterations. After staining mitochondria with TRME, various indices were calculated using the morphometric analysis software MiNA (Mitochondrial Network Analysis) [25] in order to evaluate mitochondrial morphology and to help understand functional alterations (Fig. 4C). ER stress was observed to reduce Slub voxels, which represent the area of two branches, and Junction voxels, which represent the area of three branches. Sephin1 was found to significantly reduce Slub voxels in comparison to the control group. The mitochondrial volume, as indicated by footprints, was not significantly altered by ER stress or by Sephin1 exposure. These findings are consistent with the transcript analysis and indicate that Sephin1 does not intervene in mitochondrial dynamics. Likewise, an evaluation of mitochondrial membrane potential by FACS analysis using TMRM revealed that ER stress significantly reduced membrane potential, while Sephin1 had no effect on this reduction (Fig. 4D). The impact of Sephin1 on mitochondrial quality control was evaluated through the assessment of mitophagy. Stable transfectants were generated by transfecting HK-2 with recombinant retrovirus carrying mtKeima-Red, and the percentage of positive fractions by FACS was quantified, following the exposure of the compounds for 24 h. This value demonstrated a notable increase in ER stress, while Sephin1 did not elicit a comparable change (Fig. 4E). These findings suggest that ER stress exerts a substantial influence on mitochondrial function, while Sephin1 is unlikely to directly intervene in mitochondrial function under ER stress.

### CHOP degradation by Sephin1 in HK-2 cells under ER stress

**Fig. 5:**
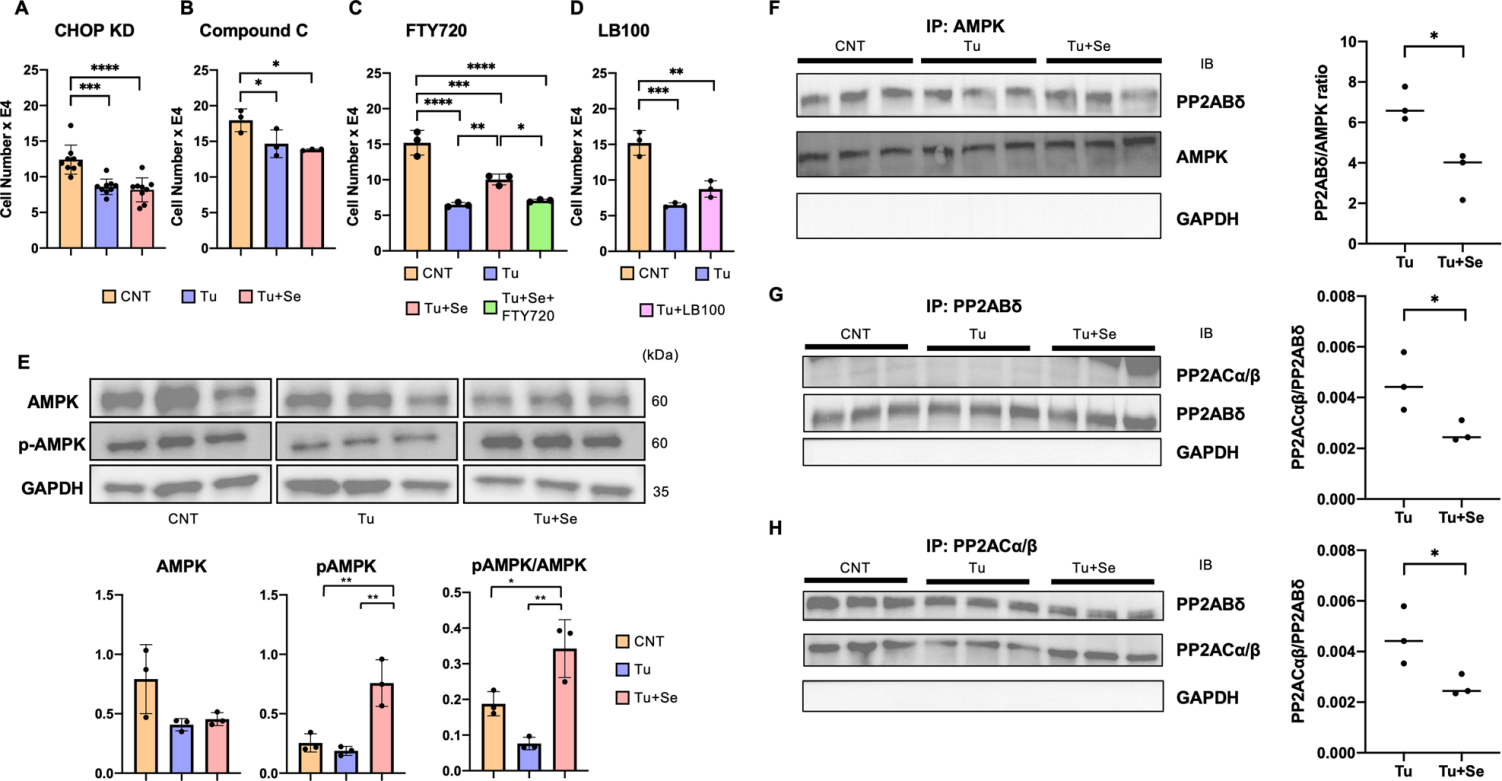
CHOP degradation by Sephin1 in HK-2 cells under ER stress. **A** Number of CHOP-KD HK-2 cells at 24 h following the administration of 20 μg/ml tunicamycin without or with 1 μM Sephin1 (*n* = 6). **B** Number of HK-2 cells in the ER stress model under 1 μM of compound C, an AMPK inhibitor (*n* = 3). **C** Number of HK-2 cells in the ER stress model to examine the effects of FTY720 at the concentration of 1 μg/ml. **D** Instead of Sephin1, LB-100, an antagonist for PP2A, was used to treat the ER stress model of HK-2 cells at the concentration of 0.01 μg/ml. **E** Western blotting analysis for AMPK and phosphorylated AMPK on Thr172 of HK-2 at 24 h following drug administration. The graph shows the expression ratio of each protein corrected by GAPDH protein expression level (*n* = 3). **F** Co-immunoprecipitation assay for AMPK and PP2ABδ. Following immunoprecipitation for AMPK, PP2ABδ was blotted for their binding status. Relative values of PP2ABδ to AMPK in tunicamycin-exposed group and Sephin1-treated group were calculated (*n* = 3). **G** Co-immunoprecipitation assay for PP2ABδ and PP2AC. Following immunoprecipitation for PP2ABδ, PP2AC was blotted for their binding status. Relative values of PP2AC to PP2ABδ in tunicamycin exposed group and Sephin1 treated group were calculated (*n* = 3). **H** Co-immunoprecipitation assay for PP2AC and PP2ABδ. Following immunoprecipitation for PP2AC, PP2ABδ was blotted for their binding status. Relative values of PP2ABδ to PP2AC in tunicamycin-exposed group and Sephin1-treated group were calculated (*n* = 3). The error bars indicate the SDs. The number of asterisks can be obtained by indicating a range of *p* values as follows; * <0.05, ** <0.01, *** <0.001, **** <0.0001.

CHOP knockdown (CHOP KD) cells were generated and used to examine the effects of Sephin1 treatment on tunicamycin exposure in these cells by transducing a recombinant lentivirus encoded with a CRISPR guide RNA against CHOP and Cas9, and clones with reduced expression of CHOP were obtained (Fig. S8A–D). Cell survival was examined by exposing CHOP KD cells to tunicamycin with or without Sephin1 (Fig. 5A). In CHOP KD cells, Sephin1 did not inhibit cell death, indicating that Sephin1 is involved in the CHOP cell death pathway. It has been reported that in the CHOP degradation pathway, phosphorylation of Ser30 is carried out by AMPK [26], which is passed on to the ubiquitin proteasome system. As shown in Fig. 4A, ER stress with tunicamycin significantly reduced the number of viable HK-2 cells, and Sephin1 treatment suppressed this reduction. In the presence of 1 μM compound C, an inhibitor of AMPK, the cell death inhibitory effect of Sephin1 was abolished (Fig. 5A). These results suggest that in the presence of Sephin1, AMPK is activated, phosphorylated and disrupts CHOP, which may contribute to the inhibition of cell death.

We hypothesized that a mechanism similar to the previously reported inhibition of PP1 formation by Sephin1 might occur in different phosphatases. AMPK is activated by Thr172 phosphorylation and inactivated by dephosphorylation of PP2A [27]. Since PP2A acquire substrate specificity by association with regulatory subunits, and their catalytic efficiency is also affected [28], we decided to investigate the involvement of Sephin1 in this pathway. We used FTY720 as an agonist against PP2A and LB-100 as an antagonist against PP2A in the HK-2 ER stress model. FTY720 at a concentration of 1 μg/ml canceled the effect of Sphin1 on cell death (Fig. 5C), while 0.01 μg/ml LB-100, used as an alternative to Sphin1, showed a tendency to inhibit cell death with tunicamycin, but did not restore cell numbers with significant differences from tunicamycin administration (Fig. 5D). These results suggest that PP2A activation dephosphorylates AMPK, leading to its inactivation, resulting in CHOP accumulation and tilting toward cell death. On the other hand, PP2A inhibition accumulates phosphorylated forms of AMPK, leading to the maintenance of AMPK activation, resulting in increased phosphorylated CHOP and tilting toward cell death inhibition. We investigated AMPK Thr172 phosphorylation under ER stress with Sephin1. The levels were not significantly altered by tunicamycin exposure but were significantly increased by nearly 4-fold by Sephin1 treatment, and the phosphorylated AMPK/AMPK ratio was also significantly increased (Fig. 5E). We first investigated how Sephin1 affects the binding of AMPK to SubgroupB isotope δ (PP2ABδ), a PP2A regulatory subunit reported to be a target of AMPK [29], by analyzing PP2AB δ expression was analyzed using CoIP (Fig. 5F, Fig. S10A). The CoIP showed that Sephin1 treatment in the ER stress state significantly attenuated the binding of AMPK to PP2ABδ. To examine PP2A holoenzyme formation under Sephin1 treatment, we performed CoIP of PP2ABδ and PP2ACα/β. Both CoIP of immunoprecipitation with PP2ABδ and immunoblotting with PP2ACα/β (Fig. 5G, Fig. S10B) and the opposite combination (Fig. 5H, Fig. S10C) showed that the association between the two proteins was significantly inhibited by Sephin1 treatment.

**Fig. 6:**
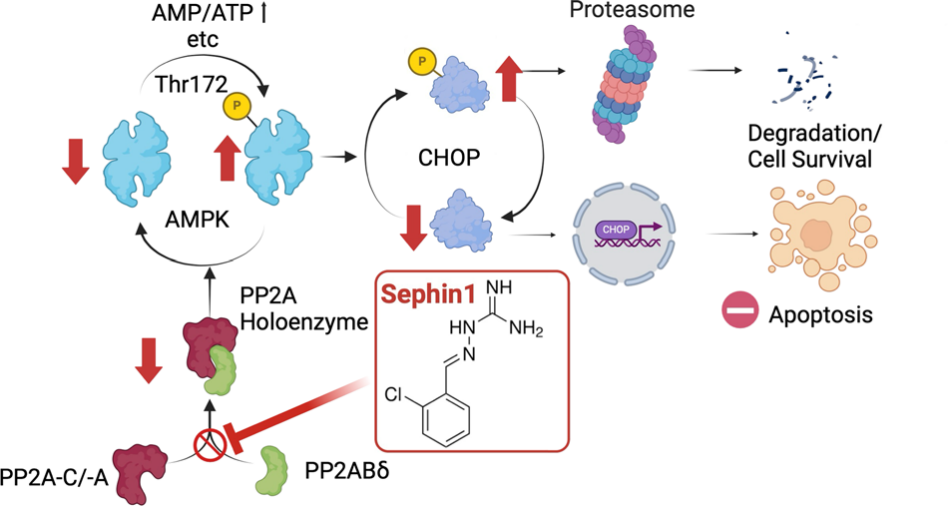
A schema showing the mechanism of action of Sephin1. Created by BioRender.

We have described a study on a new molecular target of Sephin1 and compared it with compounds reported to date to examine its potential as a CHOP inhibitor for clinical applications. Salubrinal, which acts protectively against ER stress via the PERK pathway [30], SB202190, a p38 MAPK antagonist that intervenes in the CHOP pathway [31], and vildagliptin, a dipeptidyl peptidase 4 (DPP4) inhibitor [32] were examined. Sephin1 showed a significant cytoprotective effect, while the other compounds did not produce a statistically significant cytoprotective effect in this assay. The results indicated that Sephin1 could join the development list with an advantage with regard to avoiding cell death (Fig. S10A). These results demonstrated that Sephin1 reduces pathogenesis by inhibiting cell death in the ER stress environment of the kidneys. Its survival signal is the promotion of CHOP decay by AMPK activation, and its molecular target is PP2A holoenzyme formation inhibition with PP2ABδ as a regulatory subunit (Fig. 6).

## Discussion

### Summary

In view of the doubts raised regarding the mechanism of action of Sephin1, which was developed as a holophosphatase formation inhibitor of PP1/PPP1R15A, we explored an alternative mode of action in this study. In the ER stress environment, Sephin1-mediated reduction of CHOP protein expression has been consistently documented in all previous reports [2, 5, 33], but has not been identified as a candidate mode of action of Sephin1. The present study clarifies that Sephin1 effectively attenuates the pro-apoptotic activity of CHOP through PP2A holoenzyme formation inhibition, and the previously reported neuroprotective effects can be explained by this scenario without contradiction. In a mouse model of tunicamycin-induced acute kidney injury, Sephin1 improved survival, protected tubular epithelial cells, and preserved renal function. This study showed that Sephin1 could be a potential therapeutic agent for diseases such as renal dysfunction, where cell death is central to pathophysiology.

### eIF2a phosphorylation and dephosphorylation

It was reported that Sephin1 inhibits eIF2α dephosphorylation by preventing the PP1 catalytic subunit and GADD34 from forming holophosphatase, which ameliorates neurodegenerative diseases in which ER stress is closely involved in their pathogenesis [2]. A disagreement was reported regarding the molecular mechanism of this action [5, 6]. Bio-layer interferometry showed that Sephin1 does not affect the holoenzyme formation of the PP1 catalytic subunit and GADD34, and studies using PhosTag-SDS-PAGE showed that Sephin1 does not lead to eIF2α dephosphorylation [5]. They have shown that Sephin1 suppresses CHOP expression in response to tunicamycin and histidinol (ISR inducer, not ER stress inducer) exposure in R15A KO cells and that this suppresses UPR and ISR even in eIF2α mutant cells, which do not induce UPR and ISR. The data indicating the suppression of CHOP expression by Sephin1 are consistent with the data reported in a previous report [2]. Against these rebuttions, there are reports supporting the inhibition of holophosphatase formation by Sephin1 and guanabenz, which have been reported to have a molecular mechanism of selective conformational changes in R15A [34]. This report suggests that although direct intervention of protein phosphatases, which has only a few catalytic units, is difficult, drug discovery for these protein phosphatases has great potential by targeting a large variety of regulatory subunits. However, regulatory subunits other than GADD34 have not been investigated as targets of Sephin1. Although there are conflicting claims, such studies were well-designed experimental systems, and an unified explanation of these claims would be to identify the molecular target of Sephin1 that regulates CHOP expression. We chose to approach this issue from a phenotypic perspective of cell death. ER stress induced by tunicamycin, which inhibits protein glycosylation [35] and increases misfolded protein [36], leads to cell death via the PERK pathway, depending on its extent and duration [10, 37]. One of the most prominent cell death events observed in animals treated with this drug was in the kidney in this study. It was previously reported in an acute kidney injury model using tunicamycin that cell death involves CHOP [8]. For eIF2α, we did not detect any effect on phosphorylation by Sephin1 in our experimental design (Fig. 3A). Besides the possibility that Sephin1 is not involved in the inhibition of eIF2α dephosphorylation, we cannot rule out the possibility that each pathway of ER stress is extremely dynamic and that we could not detect such changes during the study.

### CHOP as a Sephin1 target

CHOP activation is a pivotal trigger for cell death associated with ER stress [20]. Studies using CHOP-KO cells have reported that CHOP-induced cell death is central to a variety of ER stress-based pathogenesis. β cells in patients with diabetic exposed to a high demand for insulin production and secretion continue to trigger the UPR in response to this ER stress, causing β cell apoptosis in the uncompensated [38]. CHOP KO suppressed β-cell death in Akita mice, a model animal for diabetes mellitus, and improved the disease state [39]. In a streptozotocin-induced diabetes model, CHOP KO improved not only glycemic control, but also diabetic neuropathy [40]. Vildagliptin [32] and 1,2,3-triazole derivatives [41], which downregulate CHOP, protect β cells in animal studies. Inhibition of apoptosis protein-1 to promote CHOP degradation, similar to that observed in the present study, suppressed lipotoxicity in a diabetes model [42]. In neurodegenerative diseases, the involvement of CHOP in cell death has also been reported; CHOP KO inhibits amyloid β (Aβ) 1-42-induced neurodegeneration [43] and inhibited Aβ production through suppression of NF-κB binding to the β-site APP-cleaving enzyme 1 promoter [44]. It has been reported that CHOP also regulates neuronal cell death related to Parkinson’s disease [45], prion-related diseases [46], and the anesthetic sevoflurane [47]. In the kidneys, the expression of CHOP is elevated in the renal tissues of patients with chronic kidney disease [48], and 4-phenylbutyrate, which aids in protein folding, ameliorates acute kidney injury caused by tunicamycin by suppressing CHOP [49]. These reports suggest that the regulation of CHOP expression can be a target for the treatment of these diseases. In the present study, we strongly suggest that the effect of Sephin1, both in vivo and in vitro, is due to reduced CHOP levels in the nucleus. The loss of cell death inhibition effect of Sephin1 on ER stress using HK-2 CHOP KD (Fig. 5A) provides direct evidence that the point of Sephin1 action is CHOP. In addition to neurodegenerative diseases, which have been targeted previously, Sephin1 can improve renal disorders and diabetes mellitus by inhibiting cell death.

### CHOP regulation

CHOP is rarely expressed in cells under physiological conditions, but rapidly and significantly increases in the context of UPR and ISR, and it serves as a transcriptional regulator in the nucleus [50]. CHOP is regulated by controlling its expression at the transcriptional level, by suppressing protein decay in post-translational modifications [51], and by regulating nuclear migration [19]. Transcriptional regulation signals to the CHOP promoter from all three branches of the UPR that arise in response to ER stress; ATF4 downstream of PERK binds to amino acid response elements 1 (AARE1) and 2, and the C/EBP-ATF response element (CARE). Meanwhile, XBP1 and cleaved ATF6α downstream of IRE1α bind to ER stress response elements 1 (ERSE1) and 2 to drive CHOP transcription [20]. In the in vitro study using HK-2 in this study, *CHOP* mRNA was slightly changed by Sephin1. Because tunicamycin stimulates all three pathways of the UPR, the reduction of CHOP protein by Sephin1 shown in previous reports and in this study cannot be attributed to the PERK pathway eIF2α alone, suggesting that Sephin1 regulation of CHOP is not transcriptional level. The reduction of *Chop* mRNA by Sephin1 in the animal studies in this study was considered to be in the resolving phase of ER stress, considering the reduction of Grp78 and Gadd34 and the time course of the measurement, which was 3 days after exposure to tunicamycin. CHOP comprises of an N-terminal transcriptional activation/repression domain and a C-terminal basic-leucin zipper domain. Two post-translational modifications have been reported for CHOP degradation in cells: One is a serine/threonine-rich motif (97-100), which is ubiquitinated by the speckle-type POZ protein (SPOP). CHOP is thereby disrupted by the UPS [52]. Another mechanism involves the phosphorylation of Ser30 by AMPKα1, which similarly degrades CHOP in the UPS [26]. The increase in Ser30-phosphorylated CHOP by Sephin1 in mouse kidneys and HK-2 cells in this study indicates that Sephin1 intervenes in the latter regulatory pathway. The significant decrease in CHOP protein in HK-2 cells while the *Chop* mRNA by Sephin1 is invariant suggests cytoplasmic CHOP decay. The reduction of CHOP by Sephin1 in tubular epithelial cell nuclei in the mouse renal cortex, and HK-2 cell nuclei, in addition to being due to CHOP decay in the cytoplasm, also indicates the possibility of intervention in nuclear migration of CHOP. Ser107 phosphorylation of CHOP has been reported to result in binding to C/EBPβ, leading to nuclear translocation [19] and is an issue for future investigation.

### Downstream of CHOP to intrinsic and extrinsic pathway of apoptosis

The downstream effect of CHOP was first reported as the dominant negative effect of the correlating partner through binding to other C/EBP families as its mechanism of action [53]. In support of this, a genome-wide study showed that about 2/3 of the 175 genes regulated by CHOP are repressed by CHOP [54]. Subsequently, it was shown that dimers of CHOP and C/EBPβ promote isocitrate dehydrogenase1 (IDH1) transcription and cause cell death [55], and that complexes with C/EBPα activate transcription of the BH3-only protein Bim, resulting in cell death [56]. In addition, binding to ATF4 enhances cell death-related gene expression [57]. A series of studies on the inhibition of cell death by CHOP-KO have been published, revealing the dual roles of CHOP as a transcriptional activator and repressor. There are two pathways for cell death, the intrinsic/mitochondrial pathway and the extrinsic/death receptor pathway, in which CHOP acts as a regulator [20, 50]. The former pathway involves the oligomerization of BAX/BAK belonging to the BCL2 family, leading to pore formation [58], the release of cytochrome c and other proteins from mitochondria [59], and the activation of caspase3, an apoptosis executor [60]. BAX/BAK, as well as its upstream pro-apoptotic proteins, such as Bim [56] and Puma [61], are transcriptionally promoted by CHOP. Moreover, CHOP suppresses Bcl-2 and Mcl-1, which are anti-apoptotic proteins in the BLC2 family [62]. The extrinsic pathway induces apoptosis from death receptor 5 via the Fas-associated death domain to caspase8, followed by caspase3 activation [60]. This pathway is mediated by ROS [63] and ATF4 [64]. In ER stress, CHOP also plays a central role in the upregulation of this ROS. ER oxidase 1α is one of the genes whose expression is enhanced by CHOP [65]. It performs disulfide bond formation via protein disulfide isomerase and produces hydrogen peroxide as a byproduct [66]. GADD34 is also upregulated by CHOP, but dephosphorylation of eIF2α promotes resumption of translation, which in turn increases ROS [12]. Another protein upregulated by the CHOP-ATF4 heterodimer is tribbles-related protein 3 (TRB3) [67]. TRB3 inhibits anti-apoptotic activity through inhibition of AKT phosphorylation [68]. In the present study, cell death inhibition by Sephin1 is probably the latter extrinsic pathway rather than the intrinsic pathway, but direct evidence is a subject for future studies.

### AMPK regulation

AMPK signaling has been reported to be both beneficial and deleterious in amyotrophic lateral sclerosis (ALS), a neurodegenerative disease in which Sephin1 has been shown to be effective, and is still undergoing a controversial claims [69]. In studies using SOD1^G93A^, AMPK is in an overactive state [70], and administration of metformin, which activates AMPK, hastened the onset and progression of the disease [71]. On the other hand, studies using TDP-43^A315T^ have shown that the activation state of AMPK was not different from that of the wild type, whereas on the contrary, upregulation of protein phosphatase 2 A (PP2A), which inhibits AMPK activation [72]. The AMPK activators latrepirdine [73] and resveratol [74] delayed disease onset, prolonged life span, and improved disease status. A unifying understanding of these conflicting reports may be the existence of heterogeneity within the pathogenesis of ALS. Another possibility is that the compartmentalization of AMPK in cells may be critical for its function [75]. AMPK activity in cells exposed to ER stress may not depend on cell-wide AMPK phosphorylation, but rather on the dynamics of AMPK in the vicinity of the ER. Methodologies to visualize AMPK activation by site of cell presence have been reported [76] and will be a major driving force in the elucidation of the pathophysiology and development of therapeutic agents.

### PP2A regulation

The regulation of AMPK activity is activated by phosphorylation of α subunit Thr172 and inactivated by phosphorylation of α subunits Ser173, Ser496, and Thr479 [27]. PP2A, a different class of phosphatase as PP1 that was the target candidate for Sephin1, has been reported as a regulator of Thr172 phosphorylation of AMPK [28]. PP2A has a heterotrimer structure and is composed of a PP2A catalytic subunit (PP2ACα or Cβ), a PP2A A structural subunit as scaffold (PP2AAα or Aβ), and 23 classical regulatory B subunits, which are classified to 4 families (PP2AB, B’, B”, and B”’) [77]. These combinations regulate enzyme biogenesis, substrate specificity, stability, and subcellular localization [28]. LB-100, a specific antagonist of PP2A [78], and FTY720, an agonist of PP2A [79], have reached the clinic. The finding that FTY720 cancels the cytoprotective effect of Sephin1 in this study (Fig. 5C) supports that Sephin1 targets PP2A, and that LB100 did not show sufficient protective effect (Fig. 5D), in terms of PP2A inhibition may indicate the superiority of Sephin1. We have shown in this study that the molecular target of Sephin1 is PP2A holoenzyme formation inhibition with PP2ABδ as a regulatory subunit. Regarding the mode of action of Sephin1, there are currently conflicting reports on PP1 holoenzyme formation [2, 5, 6, 34], but whether other targets exist has not been reported. The present study used renal tubular cells, and future studies are needed to determine whether the mechanism of targeting PP2ABδ can be extrapolated to the mode of action of the therapeutic effect of Sephin1 on neurodegenerative diseases, which has been previously reported [2, 15]. Because the therapeutic effect of Sephin1 on neurodegenerative diseases is to inhibit cell death, and because PP2A subunits A and C are expressed ubiquitously and PP2ABδ is expressed in neurons, it is possible that the mechanism via PP2ABδ is also functioning in the nervous system. The existence of a form of ALS in which PP2A is upregulated, one of the neurodegenerative diseases for which Sephin1 has a therapeutic effect, supports the validity that one of the molecular targets of Sephin1 is PP2A holoenzyme formation [72].

### Limitations

Sphin1 was initially shown to be effective as an inhibitor of GADD34/PPP1R15A when it was first developed. In the present study, we have identified PP2ABδ as a new molecular target of Sphin1. In silico, the Protein-ligand interaction was assessed using SwissDock [80, 81] (Fig. S10B). Both the Attracting Cavity (AC) score, which represents the overall energy of the interaction, and the SwissParam score, which is designed to reproduce the binding free energy, showed a good interaction of Sephin1, with PP2ABδ being negatively larger in both scores than GADD34, suggesting a higher binding affinity than GADD34. In the present experiments, the interrelationships between Sphin1 and other than PP2ABδ were not investigated, and the fact that GADD34 has an affinity close to that of PP2ABδ in silico suggests that there may be other targets. These results indicate the need for further studies on the substrate specificity of Sephibn1. In addition, studies remain to verify in animal studies that Sephin1 targets and inhibits PP2ABδ.

## Materials and methods

### Animal experiments

C57BL/6 mice were purchased from Shimizu Laboratory Supplies Company Ltd. (Kyoto, Japan). Mice were housed in specific pathogen-free conditions with free access to food and water. Male mice aged 10-12 weeks were anesthetized by inhalation of isoflurane (099-06571, FUJIFILM Wako Pure Chemical Corp., Osaka, Japan). All experiments were performed according to the animal experiment guidelines issued by the Animal Care and Use Committee at the Kyoto Prefectural University of Medicine (Approval number M2022-530). Tunicamycin (202-08241, FUJIFILM Wako Pure Chemical Corp.) stock solutions were prepared in dimethyl sulfoxide (DMSO) (046-21981, FUJIFILM Wako Pure Chemical Corp.) adjusted to a 10 mg/ml concentration. The mice were injected with tunicamycin (1 or 2 mg/kg) or the same amount of DMSO on Day 0. Sephin1 (SML1356, Merck KGaA, Darmstadt, Germany) stock solutions were prepared in DMSO at a concentration of 10 mM. The mice were injected with Sephin1 (40 mg/kg) or the same amount of DMSO as the control group daily from Day 0 to Day 2. The mice were examined continuously for survival until Day 7. Blood samples were collected via cardiac puncture to evaluate BUN, and the hearts and kidneys were harvested for the further examinations on Day 3 (Supplementary Fig. 1A).

### Histology and immunohistochemistry

The kidneys of mice were fixed with 4% paraformaldehyde (163-20145, FUJIFILM Wako Pure Chemical Co.). The kidney tissue samples were stained with Periodic acid-Schiff (PAS) stain to quantify the kidney injury score. After deparaffinization, the paraffin sections were placed in citrate-buffered solution (pH 6.0) and boiled for 5 min to retrieve antigens. Endogenous peroxidase was quenched with 3.0% hydrogen peroxide in methanol for 20 min. Samples were blocked with 3% BSA in PBS for 30 min at room temperature and incubated with the following primary antibodies; anti-Kim-1 antibody (AF1817, R&D Systems, Minneapolis, MN, USA), anti-Megalin antibody (ab76969, Abcam plc, Cambridge, UK), or anti-CHOP antibody (sc-166682, Santa Cruz Biotechology, Inc., Dallas, Texas, USA). A goat anti-rabbit HRP-conjugated secondary antibody (414341, Nichirei Biosciences Inc., Tokyo, Japan) and 3,3’-diaminobenzidine (349-00904, FUJIFILM Wako Pure Chemical Corp.) was used for the color reaction. For the kidney injury score, five randomly selected 400x field of view photographs were taken for each kidney. Loss of brush border (LBB), cell detachment, apoptosis, cast formation, and tubular dilatation were scored. The mean value was used as the degree of injury to the kidney, and the sum of these items was calculated as the kidney injury score [16]. Tubular damage was assessed by immunohistochemistry for Megalin and Kim-1, and 10 randomly selected 400x field of view photographs were taken for one kidney, divided into the medulla and cortex. From the measurement of positive cells by a third party, the average value in one field of view was used as the score for one kidney to calculate the injury rate [82]. Nuclear translocation of CHOP was quantified in randomly selected six 400x views for each kidney.

### Cell culture

HK-2 cells (CRL-2190, American Type Culture Collection, Manassas, VA, USA) derived from human kidney were cultured in Dulbecco’s modified Eagle medium (043-30085, DMEM, FUJIFILM Wako Pure Chemical Corp.) supplemented with 10% fetal bovine serum (FBS, 10270-106, Thermo Fisher Scientific Inc., Waltham, MA, USA). Cells were incubated at 37 °C in a humidified incubator containing 5% CO_2_.

### Quantitative PCR

Total RNA from cells and tissues was extracted using TRIzol (15596018, Thermo Fisher Scientific Inc.) and a Direct-zol RNA MiniPrep Kit (R2052, Zymo Research, Irvine, CA, USA) with DNase I according to the manufacturer’s instructions. To perform qPCR, 100 ng of total RNA was reverse-transcribed using a PrimeScript RT Reagent Kit (RR036A, Takara Bio, Shiga, Japan) and a T100 thermal cycler (Bio-Rad Laboratories, Incorporated, Hercules, CA, USA). The reaction was performed using a Kapa SYBR Fast qPCR Kit Master Mix (2×) Universal (KK4602, Kapa Biosystems Ltd., Wilmington, MA, USA) on a CFX connect real-time system (Bio-Rad Laboratories, Incorporated). The relative gene expression levels were normalized to GAPDH (or Gapdh) expression.

### Apoptosis assay

We determined the apoptotic status using an Annexin V-FITC Apoptosis Detection Kit (15342-54; Nacalai Tesque Incorporated, Kyoto, Japan) according to the manufacturer’s instructions. Briefly, we harvested the cells by trypsinization and suspended them in 1×Annexin V binding solution at 1.0 × 10^6^ cells/ml. We mixed 100 μl of the cell suspension with 5 μl Annexin V-FITC solution and 5 μl propidium iodide (PI) solution and then incubated them for 15 min at room temperature in the dark. Then, we mixed the cells with 400 μl 1×Annexin V binding solution and evaluated the cells using an SH800 (Sony Corporation, Tokyo, Japan). We determined the proportion of FITC-positive apoptotic cells and PI-positive apoptotic cells using FlowJo software (663335; BD Biosciences, San Diego, CA, USA). All assays were conducted in triplicate wells and repeated at least three times in separate experiments.

### Subcellular isolation for Western blotting

HK-2 cells were resuspended in fractionation buffer (20 mM HEPES, 10 mM KCl, 2 mM MgCl_2_, 1 mM EDTA, 1 mM EGTA, 1 M DTT, 1/100 Protease Inhibitor Cocktail Set I (FUJIFILM Wako Pure Chemical Corporation), pH 7.2) and homogenized by passing 20 times through a 29-gauge needle. The lysate was maintained on ice for 20 min and then separated into pellets containing nuclei and supernatants containing the cytoplasm, membrane, and mitochondria by centrifugation at 720 × *g* for 5 min. The supernatant was centrifuged at 12,000 × *g* for 10 min. The cytoplasmic supernatant from the pellet was transferred to a clean tube. Nuclear pellets were washed with 500 μl of fractionation buffer and centrifuged again at 720 × *g* for 10 min. The pellet in fractionation buffer was resuspended and sonicated to shear genomic DNA and homogenize the lysate. These proteins were analyzed by Western blotting.

### Western blotting

The cytoplasmic protein was dissolved in RIPA buffer (182-02451, FUJIFILM Wako Pure Chemical Corporation), boiled for 10 min, electrophoresed through 10% Mini-PROTEAN TGX Precast Protein Gels (4561036, Bio-Rad Laboratories Inc.), and electroblotted onto a PVDF transfer membrane (IPVH00010, Merck KGaA). The membrane was blocked with PBS containing 5% skim milk and 0.05% Tween 20 (P1379, Merck KGaA) and incubated for 1 h with a following antibody; anti-CHOP (15204-1-AP, Proteintech Group, Inc.), anti-phospho-CHOP (PA5-104879, Thermo Fisher Scientific, Inc.), anti-EIF2A (11233-1-AP, Proteintech Group, Inc., Rosemont, IL, USA), anti-phospho-EIF2A (3398, Cell Signaling Technology, Inc., Danvers, MA, USA), anti-AMPKα (23A3) (2603, Cell Signaling Technology, Inc.), anti-phospho-AMPKα (Thr172) (40H9) (2535, Cell Signaling Technology, Inc.), anti-GAPDH (MAB374, Merck KGaA), or anti-α-tubulin (66031-1-Ig, Proteintech Group). After washing, the membrane was incubated with a 1:5000 dilution of anti-mouse IgG (7076S, Cell Signaling Technology, Incorporated) or anti-rabbit IgG HRP-linked antibody (7074S, Cell Signaling Technology, Incorporated) in blocking buffer. Subsequently, the blots were developed using Clarity Western ECL Substrate (1705060, Bio-Rad Laboratories Inc.) or Clarity Max Western ECL Substrate (1705062, Bio-Rad Laboratories Inc.), and the protein bands were visualized using a VersaDoc or ChemiDoc Imaging System (Bio-Rad Laboratories Inc.). Protein levels were quantified using ImageJ (Version 1.52).

### Immunocytochemistry

Cultured HK-2 cells were fixed in 4% paraformaldehyde at 4 °C for 15 min. in the presence of a protein-blocking solution consisting of PBS supplemented with 5% normal goat serum (X090710-8, Agilent Technologies Inc.). The cells were incubated overnight with anti-CHOP antibody (15204-1-AP, Proteintech Group, Inc.) in PBS at 4 °C. The cells were washed extensively in PBS and incubated at room temperature for 30 min with an anti-rabbit IgG (H + L) antibody tagged with Alexa Fluor 488 (Thermo Fisher Scientific, Inc.). The nuclei were counterstained with 4′,6-diamidino-2-phenylindole (DAPI; diluted 1:500, #5748, FUJIFILM Wako Pure Chemical) in PBS at room temperature for 30 min. We obtained fluorescence images using a Biorevo BZ-9000 fluorescence microscope (Keyence Corporation, Osaka, Japan).

### Co-immunoprecipitation

Interactions between various proteins were assayed using co-immunoprecipitation using Pierce Protein A/G magnetic beads (88802, Thermo Fisher Scientific Inc., MA, USA) according to the manufacturer’s instructions. Briefly, antibodies specific for PP2A subunit B (100C1, Cell Signaling Technology Inc.), PP2ABδ (116609, Gene Tex, Irvine, USA), PP2ACα/β (2259, Cell Signaling Technology Inc.), or AMPK (2603, Cell Signaling Technology, Inc.) were mixed with cell lysates in Cell Lysis Buffer, and the mixtures were incubated overnight at 4 °C. Subsequently, the mixtures were added to a microcentrifuge tube containing pre-washed protein A/G magnetic beads and incubated at 4 °C for 1 h with gentle shaking. Collected beads were placed on a magnetic stand and extensively washed to remove all unbound proteins. Proteins bound to antibodies were separated using Low-pH elution buffer. The eluted proteins were further analyzed by SDS-PAGE and Western blotting.

### Measurements of the respiratory function and glycolysis

An XFe96 extracellular flux analyzer (Agilent Technologies, Santa Clara, CA, USA) was used to measure cellular respiratory function. Cells were suspended in Seahorse XF DMEM medium (103575-100, Agilent Technologies) containing 10 mM glucose, 1 mM pyruvate, and 2 mM L-glutamine and seeded on XFe96-well microplates (101085-004, Agilent Technologies) at a density of 7 × 10^3^ cells per well. After seeding, the cells were equilibrated in a non-CO_2_ incubator for 20 min and used in the assay. For the measurement of respiratory function, oligomycin (2 μM), carbonyl cyanide p-trifluoromethoxyphenyl hydrazone (FCCP, 1 μM) and rotenone/antimycin A (0.5 μM), which were adjusted using the reagents in the Seahorse XF Cell Mito Stress Test Kit (103015-100, Agilent Technologies), were sequentially added to each well after baseline measurement. The data are presented as the oxygen consumption rate (OCR; pmol/minute). Basal respiration, ATP production, maximal respiration, proton leakage, spare respiratory capacity, non-mitochondrial oxygen (non-MTC), and coupling efficiency were calculated using Wave Controller 2.4 (Agilent Technologies). For the measurement of glycolysis, glucose (10 mM), oligomycin (1 μM), and 2-deoxy-D-glucose (2-DG, 50 mM), which were adjusted using the reagents in the Seahorse XF cell glycolysis stress test kit (103020-100, Agilent Technologies), were sequentially added to each well after baseline measurement. The data are presented as the extracellular acidification rate (ECAR; mpH/min). Glycolysis, glycolytic capacity, and glycolytic reserve were calculated using Wave Controller 2.4.

### Mitochondrial network analysis (MiNA)

We performed mitochondrial morphology analysis using the Mitochondrial Network Analysis (MiNA) toolset, which was downloaded from https://github.com/stuartlab [25]. For analysis, the image was first binarized by thresholding, assigning a maximum value of 255 to the foreground pixels and a minimum value of 0 to the background pixels. Next, using ImageJ’s built-in skeletonization function, the binary image was converted into a skeleton that represents the features of the original image as a wireframe of one-pixel-wide lines. All pixels within a skeleton were then grouped into three categories: end point voxels, slab voxels, and junction pixels. We evaluated the area of the mitochondrial footprint, slab voxels, and junction voxels of the individual cells.

### Mitochondrial membrane potential (Δφ)

The cells were resuspended at a density of 1 × 10^5^/ml in culture medium containing 100 nM Image-iT TMRM Reagent (TMRM, T668, Thermo Fisher Scientific, Incorporated) and incubated at 37 °C for 30 min. After staining, the cells were washed immediately, and resuspended in autoMACS Running Buffer (Miltenyi Biotec B.V. & Co. KG, Bergisch Gladbach, Germany), and evaluated using an SH800. The fluorescence intensity was analyzed by FlowJo.

### Mitophagy detection assay

To detect mitophagy, the pMX retroviral vector carrying Monomeric Keima Red (mKeima Red) was transfected into HK-2 cells. The mKeima Red-expressing cells were selected using SH800 1 week after retrovirus transfection. The acidic mKeima Red signal was detected using an Attune NxT Flow Cytometer (Thermo Fisher Scientific, Incorporated). mKeima Red was set at 488-nm (pH 7) and 561-nm (< pH 6) lasers with 590/40-nm and 615/20-nm emission filters, respectively. We defined the mitophagy index as the ratio of acidic (< pH 6) mKeima Red signal-positive cells to DMSO control cells at 0 h.

### Statistical analysis

The results are presented as the means ± standard deviations. The statistical significance of differences among 3 groups was evaluated using one-way ANOVA and Tukey’s multiple comparisons test, and that between 2 groups was evaluated using parametric paired t-tests for bar graphs. Mantel-Cox tests were used for statistical analysis of datasets of Kaplan‒Meier survival curves (Prism 9 software, GraphPad Prism Software Incorporated, San Diego, CA, USA). The relationship between the number of asterisks and the adjusted p-value is as follows. * (one asterisk): *p* < 0.05, ** (two asterisks): *p* < 0.01, *** (three asterisks): *p* < 0.001, **** (four asterisks): *p* < 0.0001.

## Supplementary information

Supplemental Figures

Supplemental Table

Original Western Blotting Images

## Data Availability

The raw data supporting the conclusions of this article will be made available by the authors, without undue delay.

## References

[1] Tsaytler P, Bertolotti A. Exploiting the selectivity of protein phosphatase 1 for pharmacological intervention. FEBS J. 2013;280:766–70. 22340633 10.1111/j.1742-4658.2012.08535.x

[2] Das I, Krzyzosiak A, Schneider K, Wrabetz L, D’Antonio M, Barry N, et al. Preventing proteostasis diseases by selective inhibition of a phosphatase regulatory subunit. Science. 2015;348:239–42. 25859045 10.1126/science.aaa4484 PMC4490275

[3] Costa-Mattioli M, Walter P. The integrated stress response: from mechanism to disease. Science. 2020;368:eaat5314. 32327570 10.1126/science.aat5314 PMC8997189

[4] Choy MS, Yusoff P, Lee IC, Newton JC, Goh CW, Page R, et al. Structural and Functional Analysis of the GADD34:PP1 eIF2alpha Phosphatase. Cell Rep. 2015;11:1885–91. 26095357 10.1016/j.celrep.2015.05.043 PMC4489983

[5] Crespillo-Casado A, Chambers JE, Fischer PM, Marciniak SJ, Ron D. PPP1R15A-mediated dephosphorylation of eIF2alpha is unaffected by Sephin1 or Guanabenz. Elife. 2017;6:e26109. 28447936 10.7554/eLife.26109 PMC5429092

[6] Crespillo-Casado A, Claes Z, Choy MS, Peti W, Bollen M, Ron D. A Sephin1-insensitive tripartite holophosphatase dephosphorylates translation initiation factor 2alpha. J Biol Chem. 2018;293:7766–76. 29618508 10.1074/jbc.RA118.002325 PMC5961032

[7] Ruiz A, Zuazo J, Ortiz-Sanz C, Luchena C, Matute C, Alberdi E. Sephin1 Protects Neurons against Excitotoxicity Independently of the Integrated Stress Response. Int J Mol Sci. 2020;21:6088. 32846985 10.3390/ijms21176088 PMC7504470

[8] Carlisle RE, Brimble E, Werner KE, Cruz GL, Ask K, Ingram AJ, et al. 4-Phenylbutyrate inhibits tunicamycin-induced acute kidney injury via CHOP/GADD153 repression. PLoS ONE. 2014;9:e84663. 24416259 10.1371/journal.pone.0084663 PMC3885586

[9] Gozuacik D, Bialik S, Raveh T, Mitou G, Shohat G, Sabanay H, et al. DAP-kinase is a mediator of endoplasmic reticulum stress-induced caspase activation and autophagic cell death. Cell Death Differ. 2008;15:1875–86. 18806755 10.1038/cdd.2008.121

[10] Ren J, Bi Y, Sowers JR, Hetz C, Zhang Y. Endoplasmic reticulum stress and unfolded protein response in cardiovascular diseases. Nat Rev Cardiol. 2021;18:499–521. 33619348 10.1038/s41569-021-00511-w

[11] Prola A, Nichtova Z, Pires Da Silva J, Piquereau J, Monceaux K, Guilbert A, et al. Endoplasmic reticulum stress induces cardiac dysfunction through architectural modifications and alteration of mitochondrial function in cardiomyocytes. Cardiovasc Res. 2019;115:328–42. 30084984 10.1093/cvr/cvy197

[12] Marciniak SJ, Yun CY, Oyadomari S, Novoa I, Zhang Y, Jungreis R, et al. CHOP induces death by promoting protein synthesis and oxidation in the stressed endoplasmic reticulum. Genes Dev. 2004;18:3066–77. 15601821 10.1101/gad.1250704 PMC535917

[13] Zinszner H, Kuroda M, Wang X, Batchvarova N, Lightfoot RT, Remotti H, et al. CHOP is implicated in programmed cell death in response to impaired function of the endoplasmic reticulum. Genes Dev. 1998;12:982–95. 9531536 10.1101/gad.12.7.982 PMC316680

[14] Reid DW, Tay AS, Sundaram JR, Lee IC, Chen Q, George SE, et al. Complementary roles of GADD34- and CReP-containing eukaryotic initiation factor 2alpha phosphatases during the unfolded protein response. Mol Cell Biol. 2016;36:1868–80. 27161320 10.1128/MCB.00190-16 PMC4911741

[15] Chen Y, Podojil JR, Kunjamma RB, Jones J, Weiner M, Lin W, et al. Sephin1, which prolongs the integrated stress response, is a promising therapeutic for multiple sclerosis. Brain. 2019;142:344–61. 30657878 10.1093/brain/awy322 PMC6351782

[16] Chiazza F, Chegaev K, Rogazzo M, Cutrin JC, Benetti E, Lazzarato L, et al. A nitric oxide-donor furoxan moiety improves the efficacy of edaravone against early renal dysfunction and injury evoked by ischemia/reperfusion. Oxid Med Cell Longev. 2015;2015:804659. 25834700 10.1155/2015/804659 PMC4365375

[17] Uehara M, Kusaba T, Ida T, Nakai K, Nakata T, Tomita A, et al. Pharmacological inhibition of ataxia-telangiectasia mutated exacerbates acute kidney injury by activating p53 signaling in mice. Sci Rep. 2020;10:4441. 32157166 10.1038/s41598-020-61456-7 PMC7064514

[18] Lee K, Tirasophon W, Shen X, Michalak M, Prywes R, Okada T, et al. IRE1-mediated unconventional mRNA splicing and S2P-mediated ATF6 cleavage merge to regulate XBP1 in signaling the unfolded protein response. Genes Dev. 2002;16:452–66. 11850408 10.1101/gad.964702 PMC155339

[19] Bartko JC, Li Y, Sun G, Halterman MW. Phosphorylation within the bipartite NLS alters the localization and toxicity of the ER stress response factor DDIT3/CHOP. Cell Signal. 2020;74:109713. 32673756 10.1016/j.cellsig.2020.109713 PMC7484389

[20] Yang Y, Liu L, Naik I, Braunstein Z, Zhong J, Ren B. Transcription factor C/EBP homologous protein in health and diseases. Front Immunol. 2017;8:1612. 29230213 10.3389/fimmu.2017.01612 PMC5712004

[21] Wen Q, Duan X, Liao R, Little P, Gao G, Jiang H, et al. Characterization of intracellular translocation of Forkhead transcription factor O (FoxO) members induced by NGF in PC12 cells. Neurosci Lett. 2011;498:31–36. 21549807 10.1016/j.neulet.2011.04.055

[22] Schachter TN, Shen T, Liu Y, Schneider MF. Kinetics of nuclear-cytoplasmic translocation of Foxo1 and Foxo3A in adult skeletal muscle fibers. Am J Physiol Cell Physiol. 2012;303:C977–990. 22932683 10.1152/ajpcell.00027.2012 PMC3492827

[23] Filadi R, Theurey P, Pizzo P. The endoplasmic reticulum-mitochondria coupling in health and disease: Molecules, functions and significance. Cell Calcium. 2017;62:1–15. 28108029 10.1016/j.ceca.2017.01.003

[24] Glover HL, Schreiner A, Dewson G, Tait SWG. Mitochondria and cell death. Nat Cell Biol. 2024;26:1434–1446. 38902422 10.1038/s41556-024-01429-4

[25] Valente AJ, Maddalena LA, Robb EL, Moradi F, Stuart JA. A simple ImageJ macro tool for analyzing mitochondrial network morphology in mammalian cell culture. Acta Histochem. 2017;119:315–26. 28314612 10.1016/j.acthis.2017.03.001

[26] Dai X, Ding Y, Liu Z, Zhang W, Zou MH. Phosphorylation of CHOP (C/EBP homologous protein) by the AMP-activated protein kinase alpha 1 in macrophages promotes CHOP degradation and reduces injury-induced neointimal disruption in vivo. Circ Res. 2016;119:1089–1100. 27650555 10.1161/CIRCRESAHA.116.309463 PMC5085850

[27] Steinberg GR, Hardie DG. New insights into activation and function of the AMPK. Nat Rev Mol Cell Biol. 2023;24:255–272. 10.1038/s41580-022-00547-x 36316383

[28] Hoffman A, Taleski G, Sontag E. The protein serine/threonine phosphatases PP2A, PP1 and calcineurin: a triple threat in the regulation of the neuronal cytoskeleton. Mol Cell Neurosci. 2017;84:119–31. 28126489 10.1016/j.mcn.2017.01.005

[29] Joseph BK, Liu HY, Francisco J, Pandya D, Donigan M, Gallo-Ebert C, et al. Inhibition of AMP kinase by the protein phosphatase 2A heterotrimer, PP2APpp2r2d. J Biol Chem. 2015;290:10588–98. 25694423 10.1074/jbc.M114.626259 PMC4409226

[30] Oliveira MM, Lourenco MV, Longo F, Kasica NP, Yang W, Ureta G, et al. Correction of eIF2-dependent defects in brain protein synthesis, synaptic plasticity, and memory in mouse models of Alzheimer’s disease. Sci Signal. 2021;14:eabc5429. 33531382 10.1126/scisignal.abc5429 PMC8317334

[31] Oh-Hashi K, Maruyama W, Isobe K. Peroxynitrite induces GADD34, 45, and 153 VIA p38 MAPK in human neuroblastoma SH-SY5Y cells. Free Radic Biol Med. 2001;30:213–21. 11163539 10.1016/s0891-5849(00)00461-5

[32] Hamamoto S, Kanda Y, Shimoda M, Tatsumi F, Kohara K, Tawaramoto K, et al. Vildagliptin preserves the mass and function of pancreatic β cells via the developmental regulation and suppression of oxidative and endoplasmic reticulum stress in a mouse model of diabetes. Diabetes Obes Metab. 2013;15:153–63. 22950702 10.1111/dom.12005 PMC3558804

[33] Tsaytler P, Harding HP, Ron D, Bertolotti A. Selective inhibition of a regulatory subunit of protein phosphatase 1 restores proteostasis. Science. 2011;332:91–94. 21385720 10.1126/science.1201396

[34] Carrara M, Sigurdardottir A, Bertolotti A. Decoding the selectivity of eIF2alpha holophosphatases and PPP1R15A inhibitors. Nat Struct Mol Biol. 2017;24:708–16. 28759048 10.1038/nsmb.3443 PMC5591645

[35] Yoon D, Moon JH, Cho A, Boo H, Cha JS, Lee Y, et al. Structure-based insight on the mechanism of N-glycosylation inhibition by tunicamycin. Mol Cells. 2023;46:337–44. 37190766 10.14348/molcells.2023.0001 PMC10258461

[36] Banerjee S, Ansari AA, Upadhyay SP, Mettman DJ, Hibdon JR, Quadir M, et al. Benefits and pitfalls of a glycosylation inhibitor tunicamycin in the therapeutic implication of cancers. Cells. 2024;13:395. 38474359 10.3390/cells13050395 PMC10930662

[37] Hetz C, Zhang K, Kaufman RJ. Mechanisms, regulation and functions of the unfolded protein response. Nat Rev Mol Cell Biol. 2020;21:421–38. 32457508 10.1038/s41580-020-0250-z PMC8867924

[38] Mkrtchian S. Targeting unfolded protein response in cancer and diabetes. Endocr Relat Cancer. 2015;22:C1–4. 25792543 10.1530/ERC-15-0106

[39] Oyadomari S, Koizumi A, Takeda K, Gotoh T, Akira S, Araki E, et al. Targeted disruption of the Chop gene delays endoplasmic reticulum stress-mediated diabetes. J Clin Invest. 2002;109:525–32. 11854325 10.1172/JCI14550 PMC150879

[40] Lupachyk S, Watcho P, Stavniichuk R, Shevalye H, Obrosova IG. Endoplasmic reticulum stress plays a key role in the pathogenesis of diabetic peripheral neuropathy. Diabetes. 2013;62:944–52. 23364451 10.2337/db12-0716 PMC3581201

[41] Duan H, Arora D, Li Y, Setiadi H, Xu D, Lim HY, et al. Identification of 1,2,3-triazole derivatives that protect pancreatic β cells against endoplasmic reticulum stress-mediated dysfunction and death through the inhibition of C/EBP-homologous protein expression. Bioorg Med Chem. 2016;24:2621–30. 27157393 10.1016/j.bmc.2016.03.057 PMC5176337

[42] Qi Y, Xia P. Cellular inhibitor of apoptosis protein-1 (cIAP1) plays a critical role in β-cell survival under endoplasmic reticulum stress: promoting ubiquitination and degradation of C/EBP homologous protein (CHOP). J Biol. Chem. 2012;287:32236–45. 22815481 10.1074/jbc.M112.362160 PMC3442554

[43] Baleriola J, Walker CA, Jean YY, Crary JF, Troy CM, Nagy PL, et al. Axonally synthesized ATF4 transmits a neurodegenerative signal across brain regions. Cell. 2014;158:1159–72. 25171414 10.1016/j.cell.2014.07.001 PMC4149755

[44] Marwarha G, Raza S, Prasanthi JR, Ghribi O. Gadd153 and NF-κB crosstalk regulates 27-hydroxycholesterol-induced increase in BACE1 and β-amyloid production in human neuroblastoma SH-SY5Y cells. PLoS ONE. 2013;8:e70773. 23951005 10.1371/journal.pone.0070773 PMC3739769

[45] Aimé P, Sun X, Zareen N, Rao A, Berman Z, Volpicelli-Daley L, et al. Trib3 is elevated in Parkinson’s disease and mediates death in Parkinson’s disease models. J Neurosci. 2015;35:10731–49. 26224857 10.1523/JNEUROSCI.0614-15.2015 PMC4518050

[46] Dametto P, Lakkaraju AK, Bridel C, Villiger L, O’Connor T, Herrmann US, et al. Neurodegeneration and unfolded-protein response in mice expressing a membrane-tethered flexible tail of PrP. PLoS ONE. 2015;10:e0117412. 25658480 10.1371/journal.pone.0117412 PMC4319788

[47] Liu B, Xia J, Chen Y, Zhang J. Sevoflurane-induced endoplasmic reticulum stress contributes to neuroapoptosis and BACE-1 expression in the developing brain: the role of eIF2α. Neurotox Res. 2017;31:218–29. 27682474 10.1007/s12640-016-9671-z

[48] Wu X, He Y, Jing Y, Li K, Zhang J. Albumin overload induces apoptosis in renal tubular epithelial cells through a CHOP-dependent pathway. Omics. 2010;14:61–73. 20141329 10.1089/omi.2009.0073

[49] Mohammed-Ali Z, Lu C, Marway MK, Carlisle RE, Ask K, Lukic D, et al. Endoplasmic reticulum stress inhibition attenuates hypertensive chronic kidney disease through reduction in proteinuria. Sci Rep. 2017;7:41572. 28148966 10.1038/srep41572 PMC5288651

[50] Hu H, Tian M, Ding C, Yu S. The C/EBP homologous protein (CHOP) transcription factor functions in endoplasmic reticulum stress-induced apoptosis and microbial infection. Front Immunol. 2018;9:3083. 30662442 10.3389/fimmu.2018.03083 PMC6328441

[51] Ohoka N, Hattori T, Kitagawa M, Onozaki K, Hayashi H. Critical and functional regulation of CHOP (C/EBP homologous protein) through the N-terminal portion. J Biol Chem. 2007;282:35687–94. 17872950 10.1074/jbc.M703735200

[52] Zhang P, Gao K, Tang Y, Jin X, An J, Yu H, et al. Destruction of DDIT3/CHOP protein by wild-type SPOP but not prostate cancer-associated mutants. Hum Mutat. 2014;35:1142–51. 24990631 10.1002/humu.22614

[53] Ron D, Habener JF. CHOP, a novel developmentally regulated nuclear protein that dimerizes with transcription factors C/EBP and LAP and functions as a dominant-negative inhibitor of gene transcription. Genes Dev. 1992;6:439–53. 1547942 10.1101/gad.6.3.439

[54] Jauhiainen A, Thomsen C, Strombom L, Grundevik P, Andersson C, Danielsson A, et al. Distinct cytoplasmic and nuclear functions of the stress induced protein DDIT3/CHOP/GADD153. PLoS ONE. 2012;7:e33208. 22496745 10.1371/journal.pone.0033208 PMC3322118

[55] Yang X, Du T, Wang X, Zhang Y, Hu W, Du X, et al. IDH1, a CHOP and C/EBPbeta-responsive gene under ER stress, sensitizes human melanoma cells to hypoxia-induced apoptosis. Cancer Lett. 2015;365:201–10. 26049021 10.1016/j.canlet.2015.05.027

[56] Puthalakath H, O’Reilly LA, Gunn P, Lee L, Kelly PN, Huntington ND, et al. ER stress triggers apoptosis by activating BH3-only protein Bim. Cell. 2007;129:1337–49. 17604722 10.1016/j.cell.2007.04.027

[57] Han J, Back SH, Hur J, Lin YH, Gildersleeve R, Shan J, et al. ER-stress-induced transcriptional regulation increases protein synthesis leading to cell death. Nat Cell Biol. 2013;15:481–90. 23624402 10.1038/ncb2738 PMC3692270

[58] Moldoveanu T, Czabotar PE. BAX, BAK, and BOK: a coming of age for the BCL-2 family effector proteins. Cold Spring Harb Perspect Biol. 2020;12:a036319. 10.1101/cshperspect.a036319 PMC7111251 31570337

[59] Tuzlak S, Kaufmann T, Villunger A. Interrogating the relevance of mitochondrial apoptosis for vertebrate development and postnatal tissue homeostasis. Genes Dev. 2016;30:2133–51. 27798841 10.1101/gad.289298.116 PMC5088563

[60] Yuan J, Ofengeim D. A guide to cell death pathways. Nat Rev Mol Cell Biol. 2024;25:379–95. 38110635 10.1038/s41580-023-00689-6

[61] Galehdar Z, Swan P, Fuerth B, Callaghan SM, Park DS, Cregan SP. Neuronal apoptosis induced by endoplasmic reticulum stress is regulated by ATF4-CHOP-mediated induction of the Bcl-2 homology 3-only member PUMA. J Neurosci. 2010;30:16938–48. 21159964 10.1523/JNEUROSCI.1598-10.2010 PMC6634926

[62] Kale J, Osterlund EJ, Andrews DW. BCL-2 family proteins: changing partners in the dance towards death. Cell Death Differ. 2018;25:65–80. 29149100 10.1038/cdd.2017.186 PMC5729540

[63] Chang CC, Kuan CP, Lin JY, Lai JS, Ho TF. Tanshinone IIA facilitates TRAIL sensitization by up-regulating DR5 through the ROS-JNK-CHOP signaling axis in human ovarian carcinoma cell lines. Chem Res Toxicol. 2015;28:1574–83. 26203587 10.1021/acs.chemrestox.5b00150

[64] Chen P, Hu T, Liang Y, Li P, Chen X, Zhang J, et al. Neddylation inhibition activates the extrinsic apoptosis pathway through ATF4-CHOP-DR5 axis in human esophageal cancer cells. Clin Cancer Res. 2016;22:4145–57. 26983464 10.1158/1078-0432.CCR-15-2254

[65] Rao J, Zhang C, Wang P, Lu L, Qian X, Qin J, et al. C/EBP homologous protein (CHOP) contributes to hepatocyte death via the promotion of ERO1α signalling in acute liver failure. Biochem J. 2015;466:369–78. 25387528 10.1042/BJ20140412

[66] Ramming T, Okumura M, Kanemura S, Baday S, Birk J, Moes S, et al. A PDI-catalyzed thiol-disulfide switch regulates the production of hydrogen peroxide by human Ero1. Free Radic Biol Med. 2015;83:361–72. 25697776 10.1016/j.freeradbiomed.2015.02.011

[67] Morse E, Schroth J, You YH, Pizzo DP, Okada S, Ramachandrarao S, et al. TRB3 is stimulated in diabetic kidneys, regulated by the ER stress marker CHOP, and is a suppressor of podocyte MCP-1. Am J Physiol Renal Physiol. 2010;299:F965–972. 20660016 10.1152/ajprenal.00236.2010 PMC2980398

[68] Du K, Herzig S, Kulkarni RN, Montminy M. TRB3: a tribbles homolog that inhibits Akt/PKB activation by insulin in liver. Science. 2003;300:1574–7. 12791994 10.1126/science.1079817

[69] Perera ND, Turner BJ. AMPK signalling and defective energy metabolism in amyotrophic lateral sclerosis. Neurochem Res. 2016;41:544–53. 26202426 10.1007/s11064-015-1665-3

[70] Sui Y, Zhao Z, Liu R, Cai B, Fan D. Adenosine monophosphate-activated protein kinase activation enhances embryonic neural stem cell apoptosis in a mouse model of amyotrophic lateral sclerosis. Neural Regen Res. 2014;9:1770–8. 25422638 10.4103/1673-5374.143421 PMC4238165

[71] Kaneb HM, Sharp PS, Rahmani-Kondori N, Wells DJ. Metformin treatment has no beneficial effect in a dose-response survival study in the SOD1(G93A) mouse model of ALS and is harmful in female mice. PLoS ONE. 2011;6:e24189. 21909419 10.1371/journal.pone.0024189 PMC3164704

[72] Perera ND, Sheean RK, Scott JW, Kemp BE, Horne MK, Turner BJ. Mutant TDP-43 deregulates AMPK activation by PP2A in ALS models. PLoS ONE. 2014;9:e95549. 24595038 10.1371/journal.pone.0090449 PMC3942426

[73] Coughlan KS, Mitchem MR, Hogg MC, Prehn JH. “Preconditioning” with latrepirdine, an adenosine 5’-monophosphate-activated protein kinase activator, delays amyotrophic lateral sclerosis progression in SOD1(G93A) mice. Neurobiol Aging. 2015;36:1140–50. 25443289 10.1016/j.neurobiolaging.2014.09.022

[74] Mancuso R, del Valle J, Modol L, Martinez A, Granado-Serrano AB, Ramirez-Núñez O, et al. Resveratrol improves motoneuron function and extends survival in SOD1(G93A) ALS mice. Neurotherapeutics. 2014;11:419–32. 24414863 10.1007/s13311-013-0253-y PMC3996124

[75] Chauhan AS, Zhuang L, Gan B. Spatial control of AMPK signaling at subcellular compartments. Crit Rev Biochem Mol Biol. 2020;55:17–32. 32069425 10.1080/10409238.2020.1727840 PMC8237692

[76] Miyamoto T, Rho E, Sample V, Akano H, Magari M, Ueno T, et al. Compartmentalized AMPK signaling illuminated by genetically encoded molecular sensors and actuators. Cell Rep. 2015;11:657–70. 25892241 10.1016/j.celrep.2015.03.057 PMC4417068

[77] Lubbers ER, Mohler PJ. Roles and regulation of protein phosphatase 2A (PP2A) in the heart. J Mol Cell Cardiol. 2016;101:127–33. 27832939 10.1016/j.yjmcc.2016.11.003 PMC5939568

[78] Li XF, Li SY, Dai CM, Li JC, Huang DR, Wang JY. PP2A inhibition by LB-100 protects retinal pigment epithelium cells from UV radiation via activation of AMPK signaling. Biochem Biophys Res Commun. 2018;506:73–80. 30340831 10.1016/j.bbrc.2018.10.077

[79] Zhong Y, Tian F, Ma H, Wang H, Yang W, Liu Z, et al. FTY720 induces ferroptosis and autophagy via PP2A/AMPK pathway in multiple myeloma cells. Life Sci. 2020;260:118077. 32810509 10.1016/j.lfs.2020.118077

[80] Bugnon M, Rohrig UF, Goullieux M, Perez MAS, Daina A, Michielin O, et al. SwissDock 2024: major enhancements for small-molecule docking with Attracting Cavities and AutoDock Vina. Nucleic Acids Res. 2024;52:W324–W332. 38686803 10.1093/nar/gkae300 PMC11223881

[81] Rohrig UF, Goullieux M, Bugnon M, Zoete V. Attracting cavities 2.0: improving the flexibility and robustness for small-molecule docking. J Chem Inf Model. 2023;63:3925–40. 37285197 10.1021/acs.jcim.3c00054 PMC10305763

[82] Yamashita N, Nakai K, Nakata T, Nakamura I, Kirita Y, Matoba S, et al. Cumulative DNA damage by repeated low-dose cisplatin injection promotes the transition of acute to chronic kidney injury in mice. Sci Rep. 2021;11:20920. 34686727 10.1038/s41598-021-00392-6 PMC8536734

