# Supplementary material for: Sephin1 suppresses ER stress-induced cell death by inhibiting the formation of PP2A holoenzyme
Source: Cell Death Dis. 2025 Feb 19;16(1):117. doi: 10.1038/s41419-025-07450-1 (PMC11840111; doi:10.1038/s41419-025-07450-1)
Supplement: Supplementary file 1 — Supplemental Figures [file 41419_2025_7450_MOESM1_ESM.pdf]

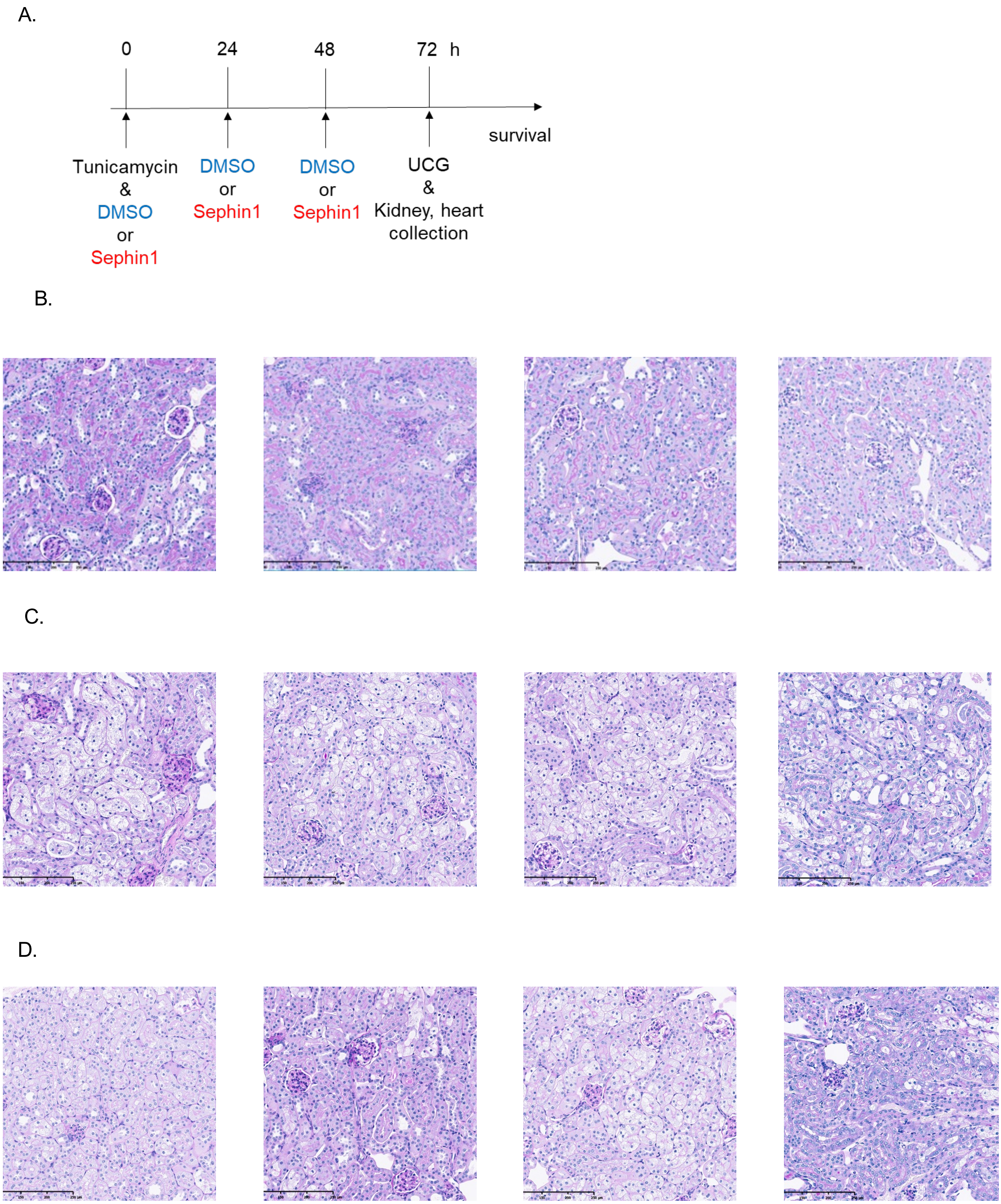

Supplementary Figure 1

A: Time course of the animal experiments. C57BL/6 mice were intraperitoneally treated with tunicamycin and DMSO or Sephin1 every 24 h.

B-D: PAS staining images to be chosen to evaluate kidney injury scores in control group(B), in tunicamycin group (C), and in tunicamycin + Sephin1 group (D).

A.

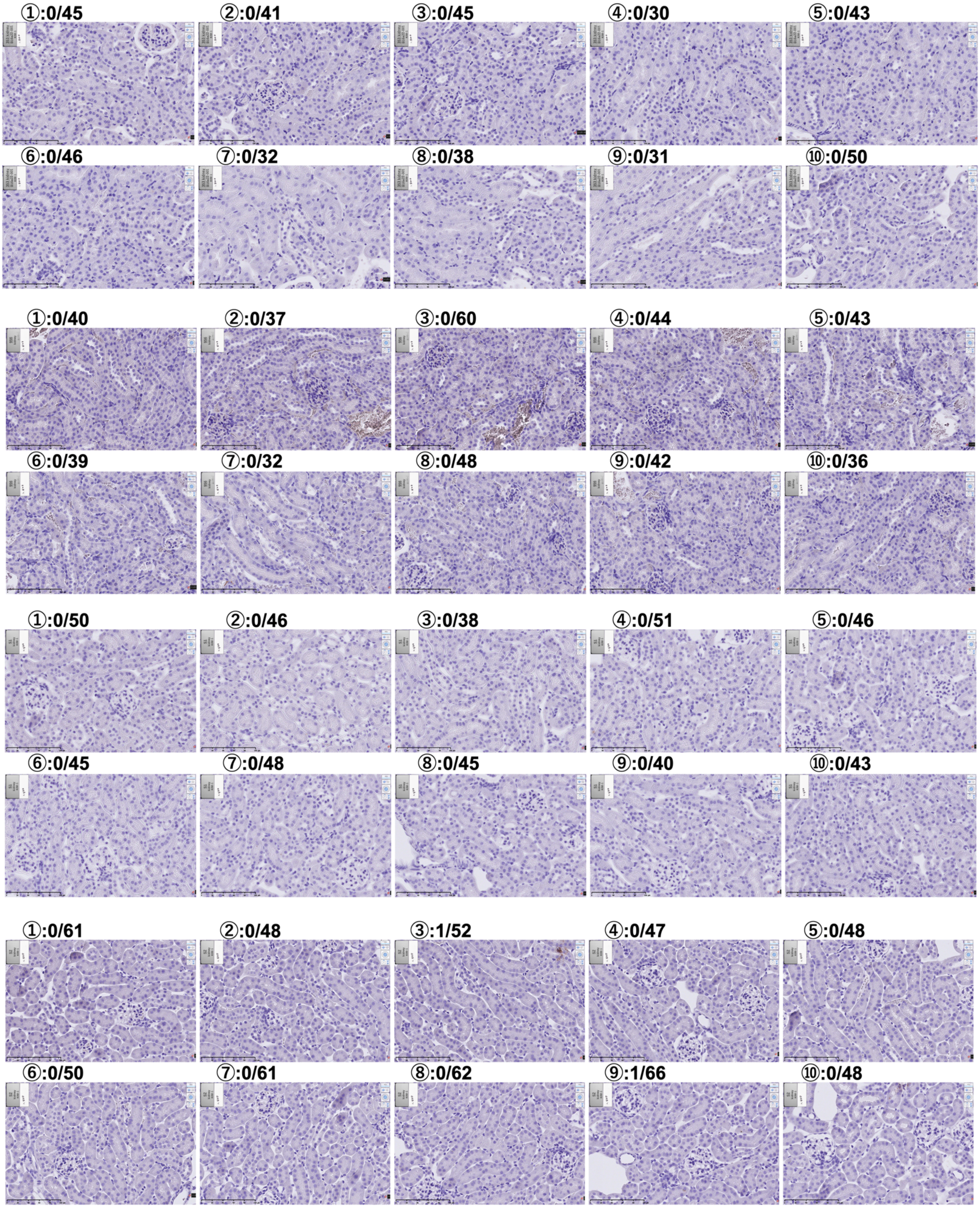

Supplementary Figure 2

A: Megalin and Kim-1 staining images to be quantify the damaged tubules in the control group

B.

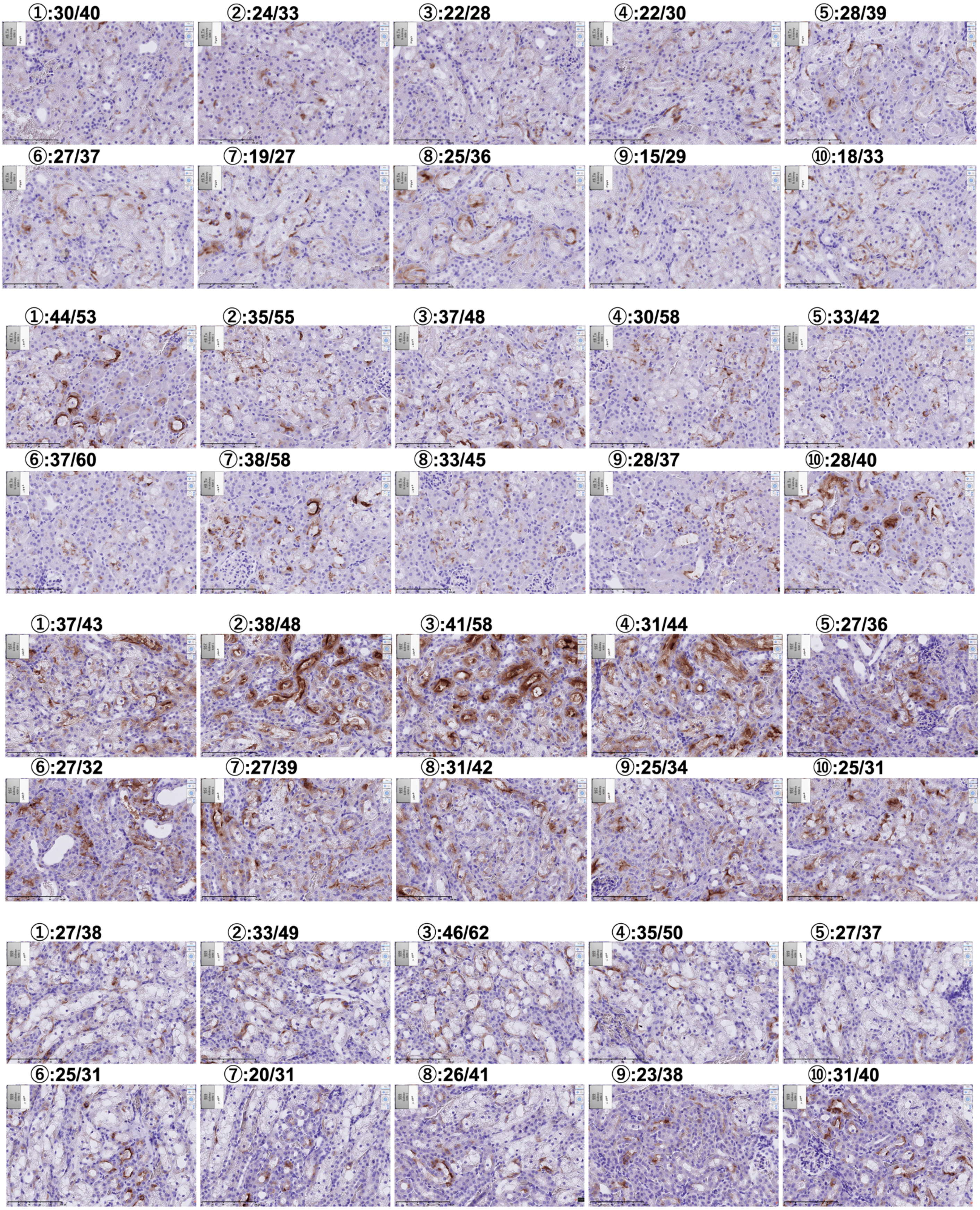

Supplementary Figure 2

B: Megalin and Kim-1 staining images to be quantify the damaged tubules in tunicamycin group

C.

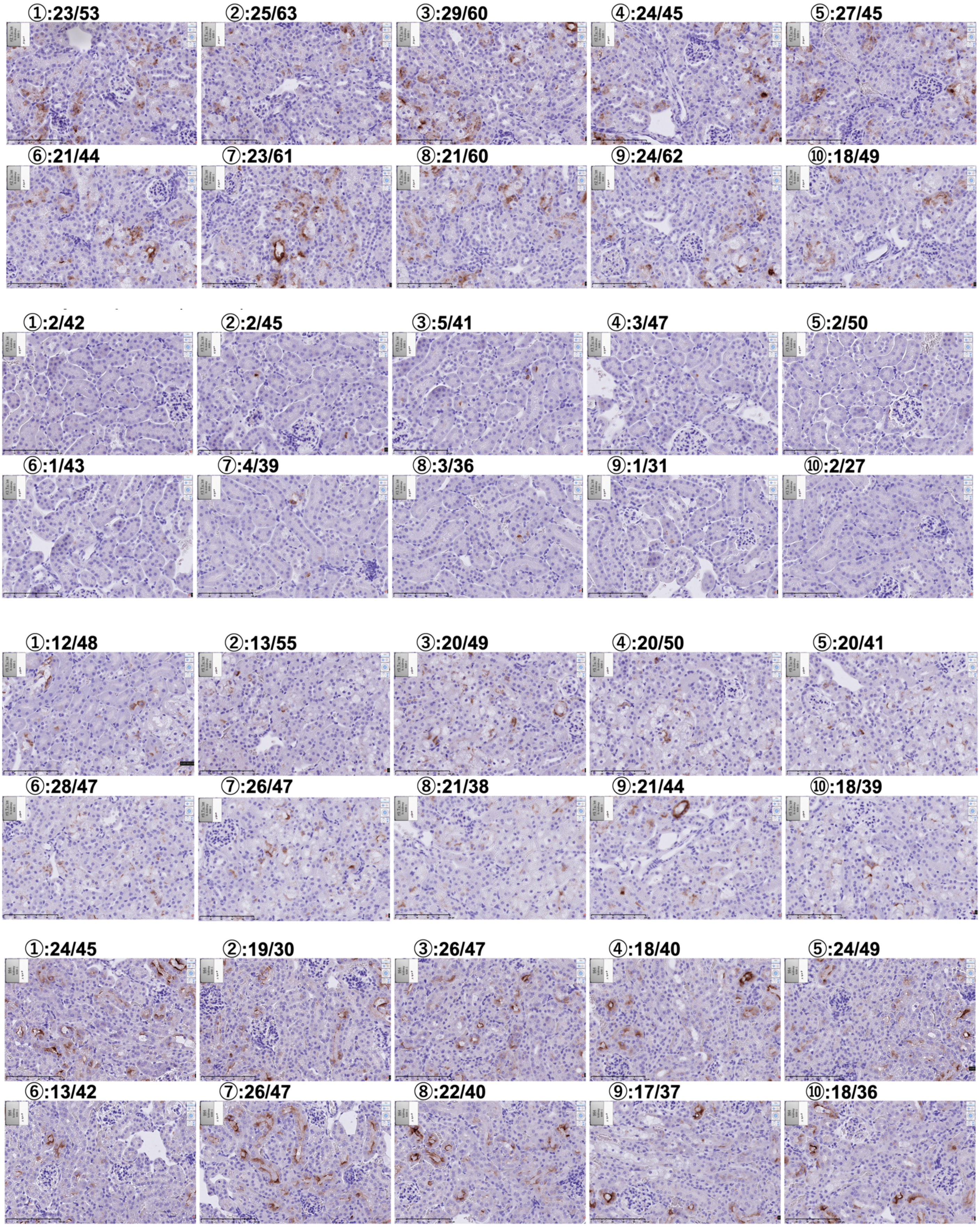

Supplementary Figure 2

C: Megalin and Kim-1 staining images to be quantify the damaged tubules in tunicamycin + Sephin1 group.

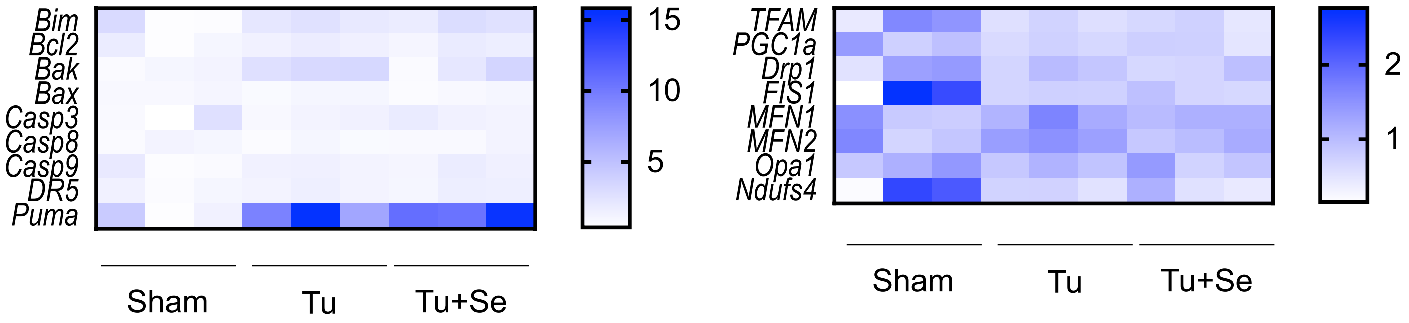

Supplementary Figure 3

Heat map of mRNA expression in murine kidney on Day 3

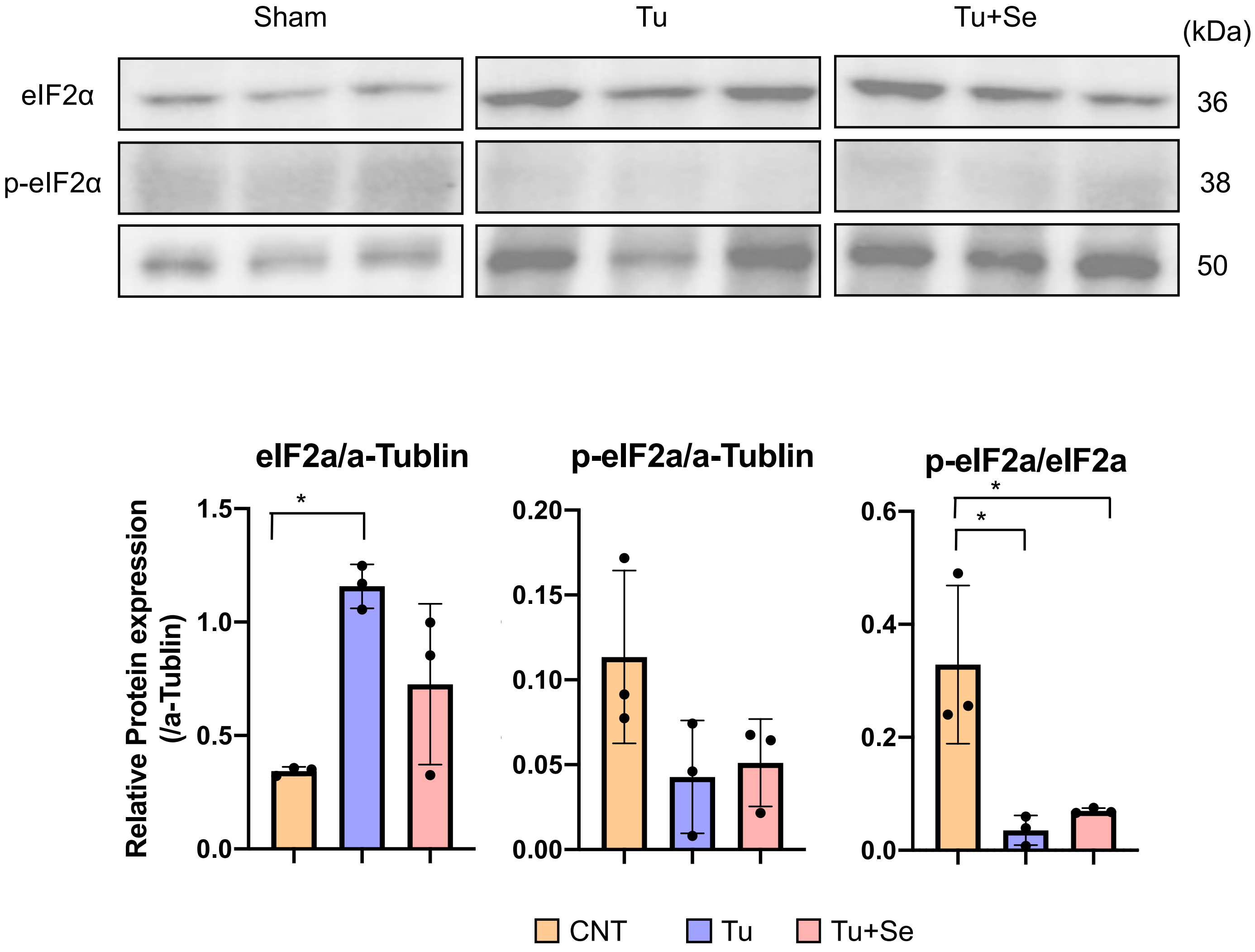

Supplementary Figure. 4

Western blot analysis of eIF2α of murine kidney on Day 3. The graph shows the expression ratio of each protein corrected by α-Tubulin protein expression level (n = 3).

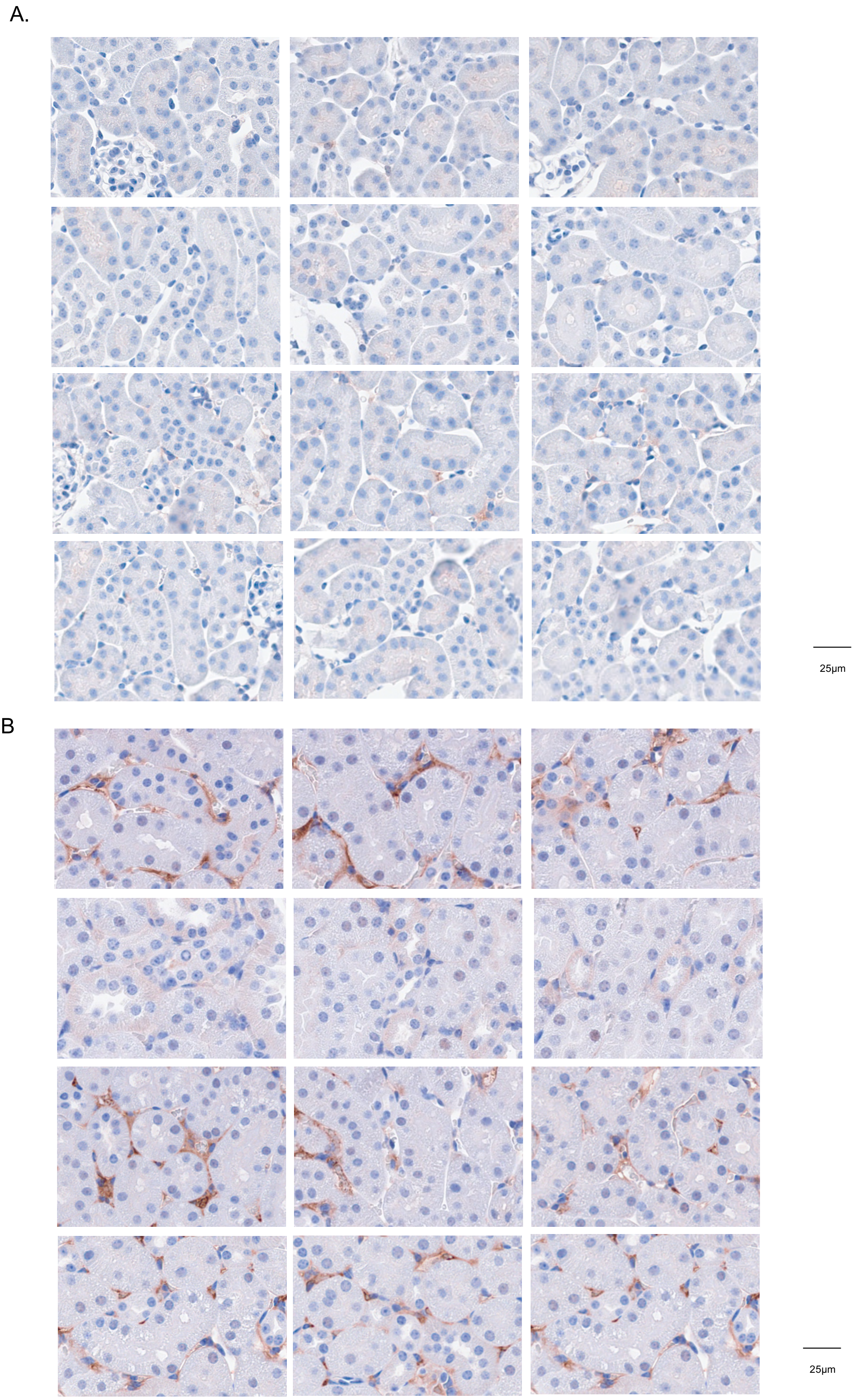

Supplementary Figure 5

Immunohistochemistry for CHOP in the kidney in the control group (A), tunicamycin group (B)

C.

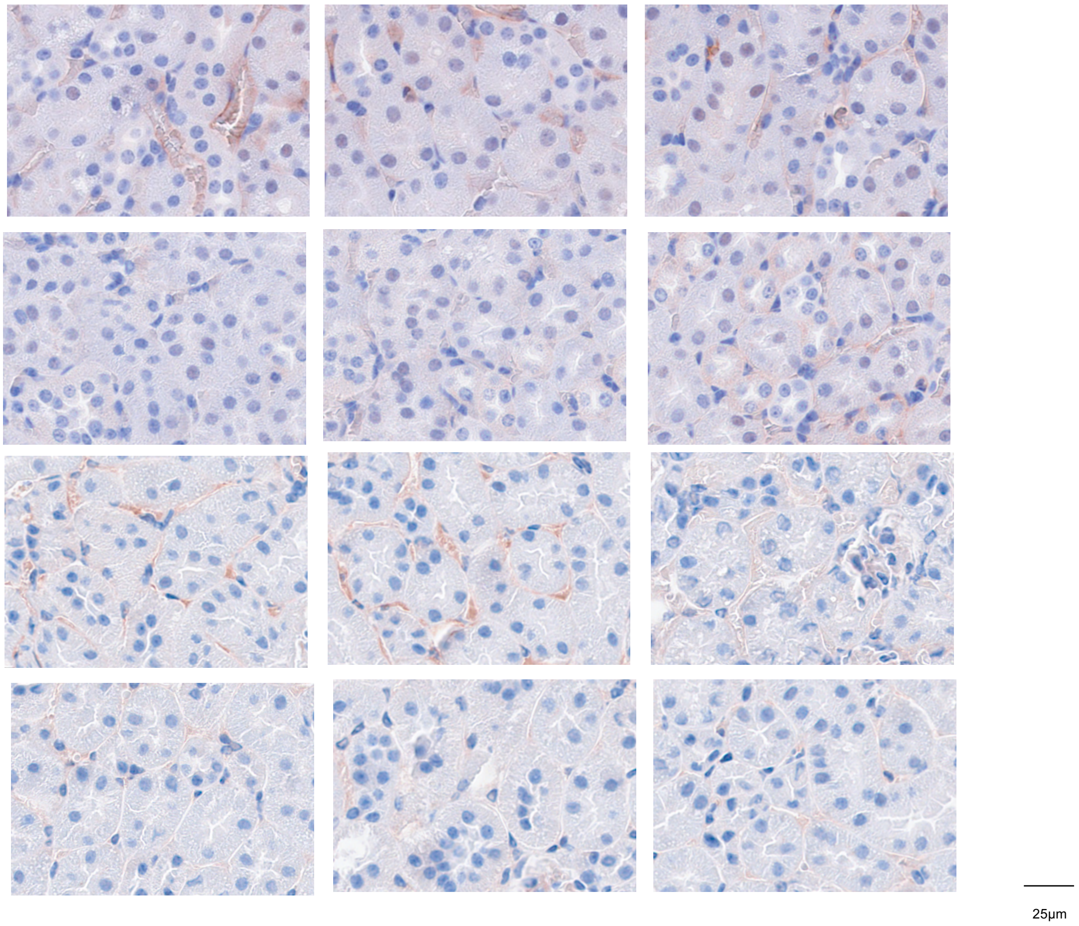

Supplementary Figure 5

Immunohistochemistry for CHOP in either the cortex or outer medulla of the kidney in tunicamycin + Sephin1 group **(C)**.

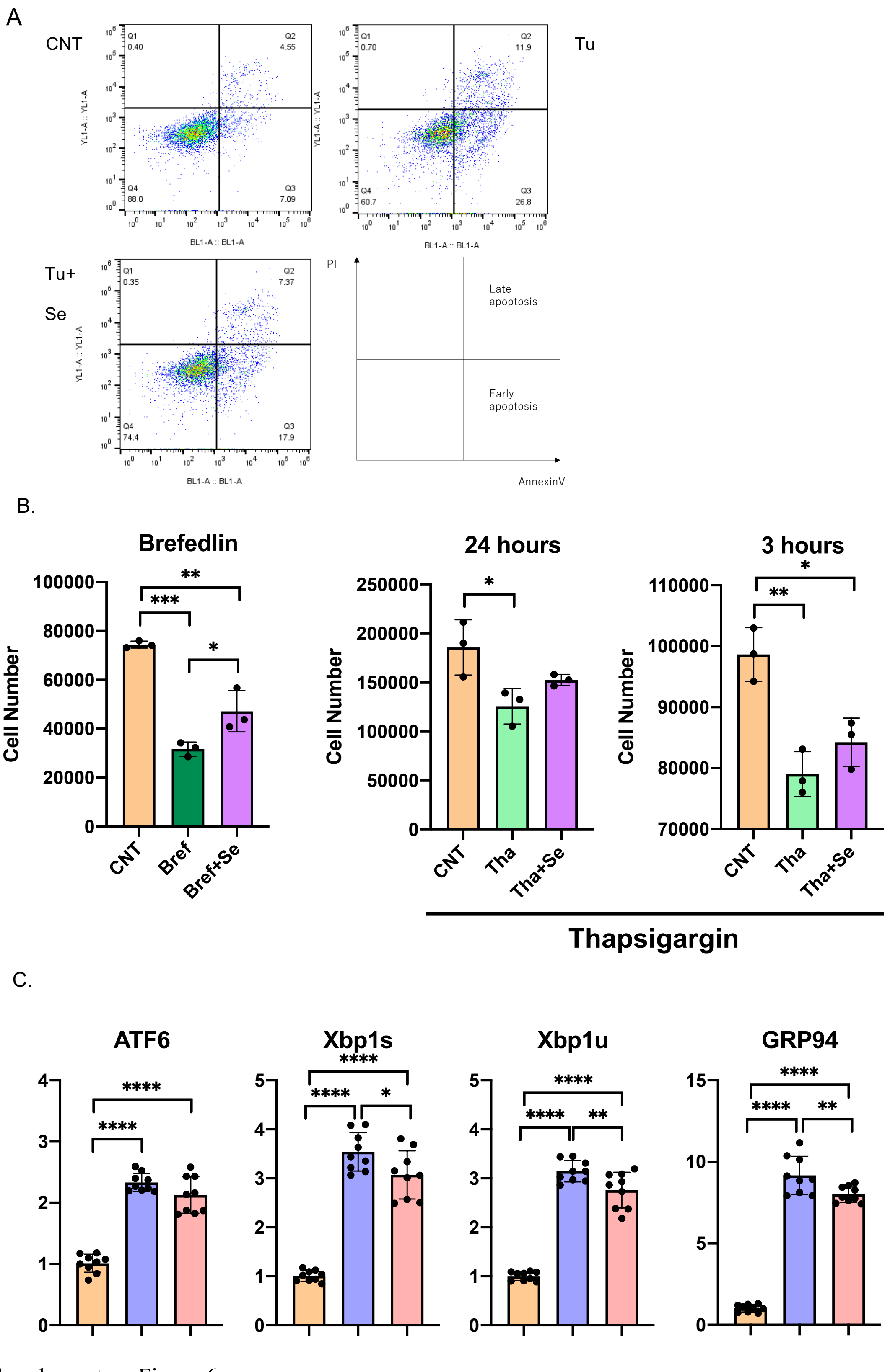

Supplementary Figure 6

A: Representative FACS dot blot analysis images for apoptosis assay

B: Effect of Sephin1 on ER stressors other than tunicamycin. Brefedlin was added to the HK-2 culture medium at 5uM and thapsigargin at a concentration of 300nM, replacing tunicamycin, while Sephin1 was added simultaneously at a concentration of 1uM.

C: The mRNA expression of HK-2 cells at 24 h following the administration of 20 μ g/ml tunicamycin without or with 1 μ M Sephin1, Key factors in the ATF6 and IRE1 pathways other than PERK in the UPR, and a main chaperone (n = 3).

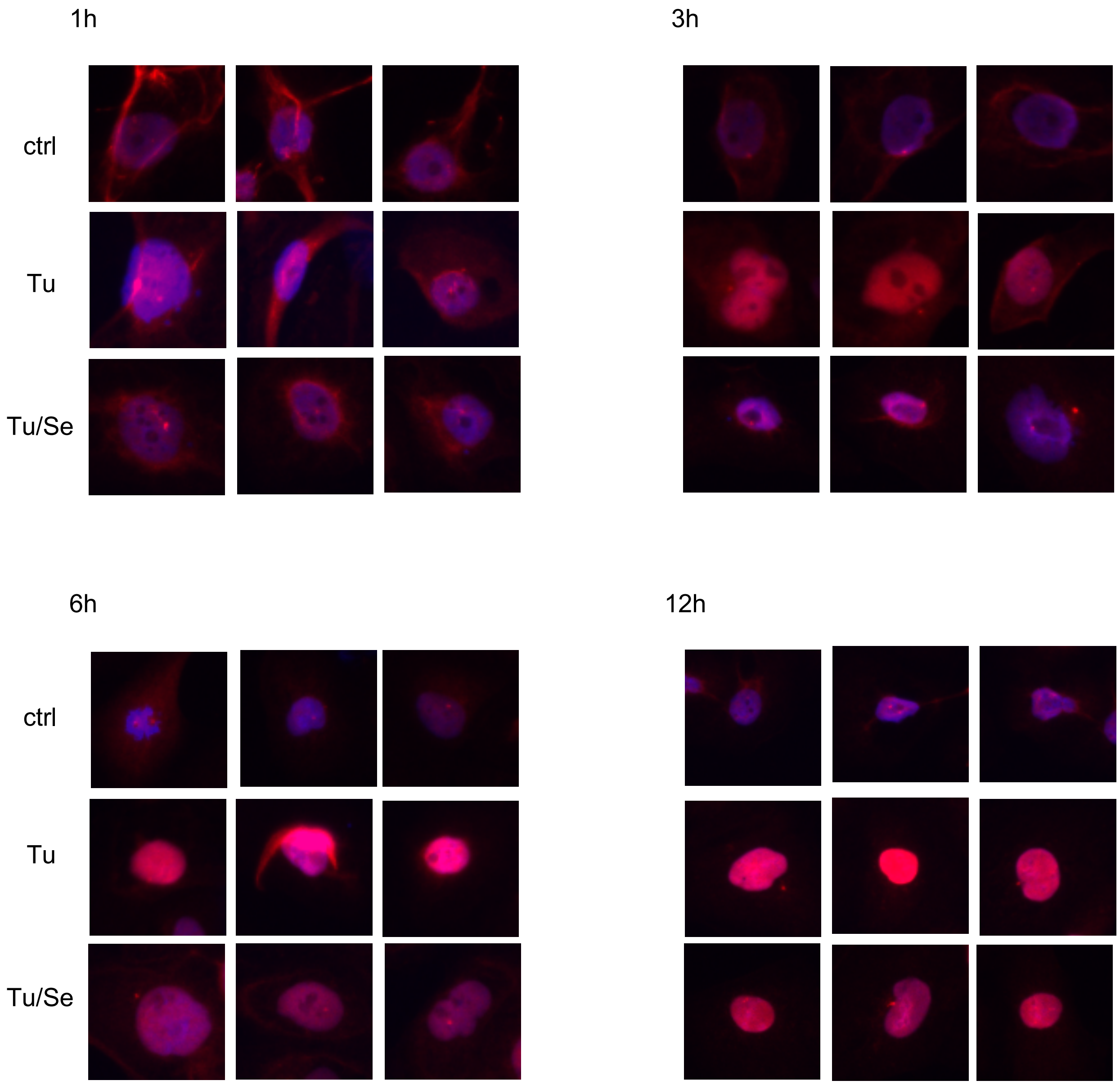

Supplementary Figure 7

Representative 3 images of immunocytochemistry for CHOP in the nucleus over time following tunicamycin administration with or without Sephin1.

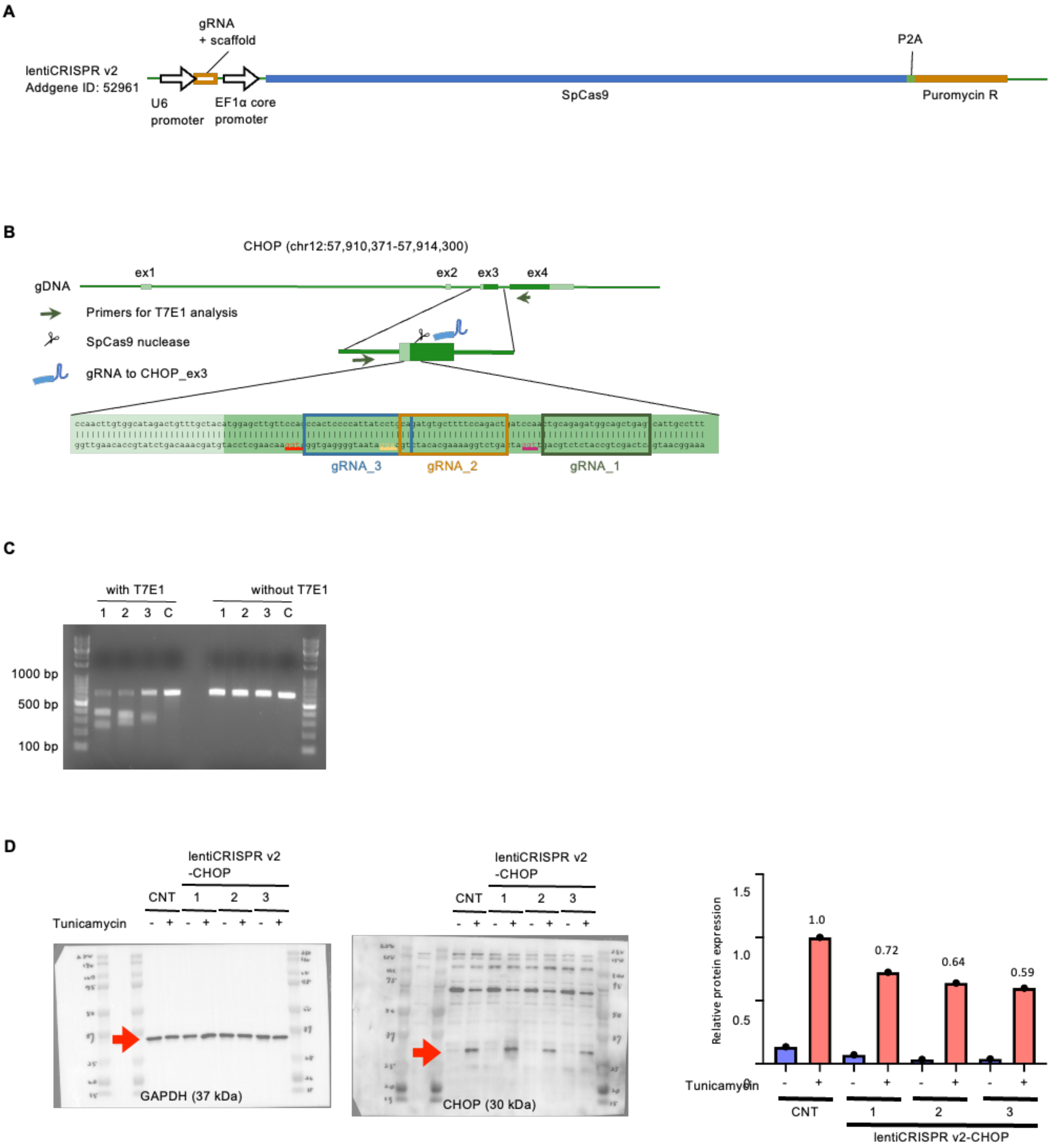

Supplementary Figure 8

Generation of CHOP knockdown HK-2 cells. (A) Recombinant lentiCRISPR map carrying SpCas9 and gDNA. (B) DNA sequence information of gRNA targeting CHOP exon 3. (C) T7E1 assay to verify the mutation induction efficacy in three independent experiments. (D) Western blot images of gene-transduced cells for CHOP expression, and a bar graph showing the reduction rate of CHOP. For the ER stress model, #3 cells were used to evaluate CHOP involvement following Sephihn1 administration.

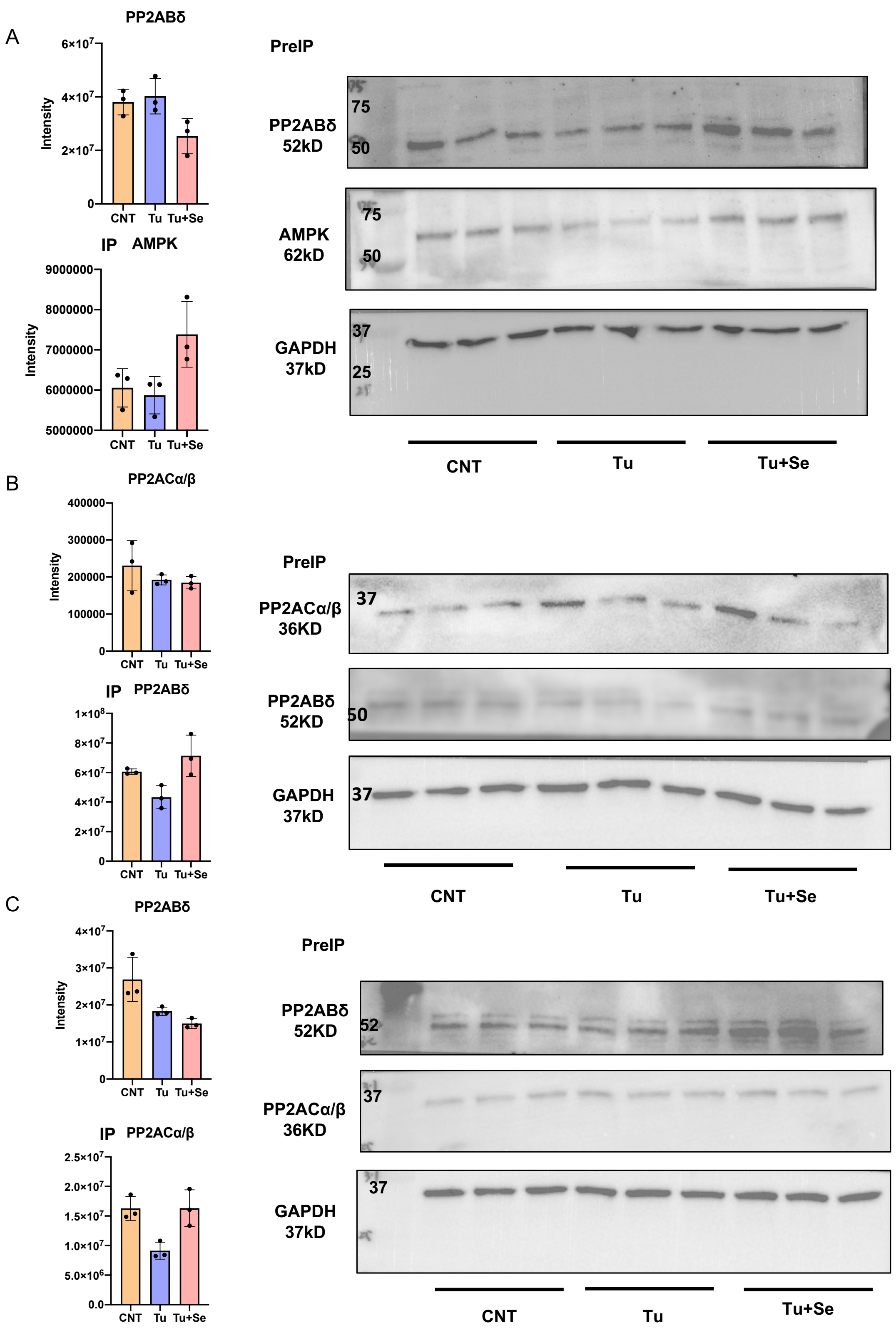

Supplementary Figure 9

A: Left: Quantification of WB of AMPK and PP2AB δ against AMPK immunoprecipitates. Right: WB for each protein before CoIP. N=3

B: Left: Quantification of WB of PP2AB δ and PP2AC α / β against PP2AB δ immunoprecipitates. Right: WB for each protein before CoIP. N=3

C: Left: Quantification of WB of PP2AC α / β and PP2AB δ against PP2AC α / β immunoprecipitates. Right: WB for each protein before CoIP. N=3

A.

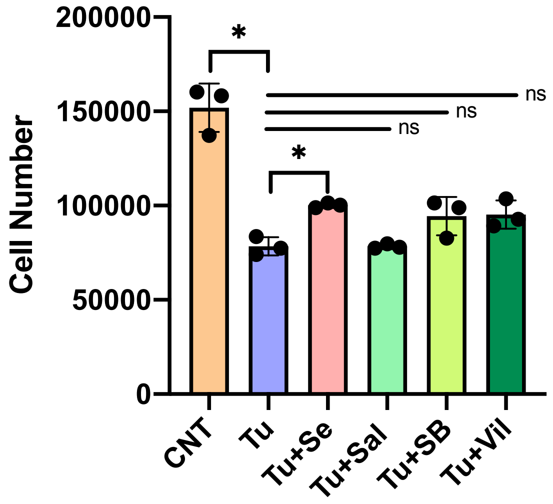

B.

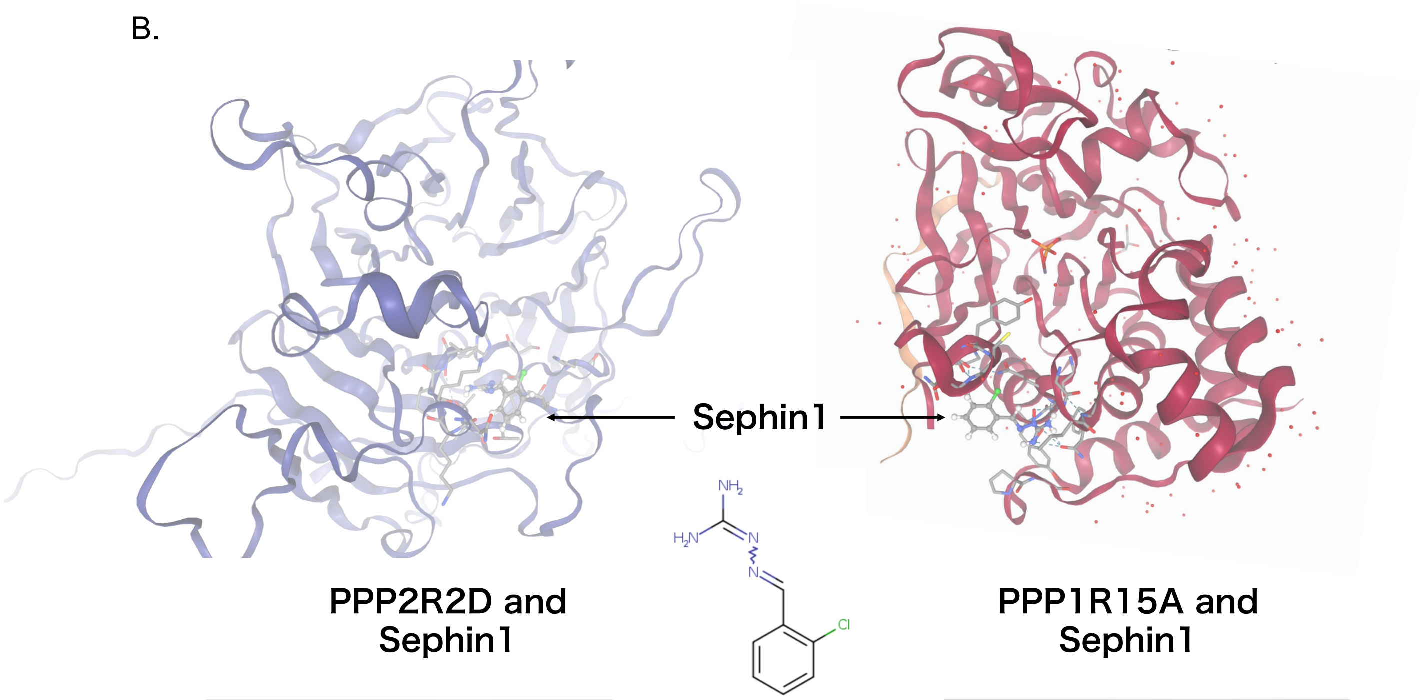

AC Score	SwissParam Score
-74.641554	-6.7157

AC Score	SwissParam Score
-71.217410	-6.4146

Supplementary Figure 10

A: Comparison of the effect of Sephin1 on tunicamycin-induced ER stress on HK-2 cells with that of other reported CHOP inhibitors. All compounds were added at a concentration of 2 μM. N=3

B: In silico docking simulation of protein ligand interaction of Sephin1 with PP2AB δ and GADD34 by SwissDock.
