# Supplementary material for: Sephin1 suppresses ER stress-induced cell death by inhibiting the formation of PP2A holoenzyme
Source: Cell Death Dis. 2025 Feb 19;16(1):117. doi: 10.1038/s41419-025-07450-1 (PMC11840111; doi:10.1038/s41419-025-07450-1)
Supplement: Supplementary file 2 — Supplemental Table [file 41419_2025_7450_MOESM2_ESM.docx]

| Role | Class | Isoform | Alternative name | Gene |
| --- | --- | --- | --- | --- |
| Scaffolding | A | PP2AAα | PR65A | PPP2R1A |
|  |  | PP2AAβ | PR65B | PPP2R1B |
| Regulatory | B | PP2ABα | PR55α, B55α | PPP2R2A |
|  |  | PP2ABβ | PR55β, Β55β | PPP2R2B |
|  |  | PP2ABγ | PR55γ, Β55γ | PPP2R2C |
|  |  | PP2ABδ | PR55δ, Β55δ | PPP2R2D |
|  | B' | PP2AB'α | PR61α, Β56α | PPP2R5A |
|  |  | PP2AB'β | PR61β, Β56β | PPP2R5B |
|  |  | PP2AB'γ | PR61γ, Β56γ | PPP2R5C |
|  |  | PP2AB'δ | PR61δ, Β56δ | PPP2R5D |
|  |  | PP2AB'ε | PR61ε, Β56ε | PPP2R5E |
|  | B'' | PP2AB''α | PR72, PR130 | PPP2R3A |
|  |  | PP2AB''β | PR48, PR70 | PPP2R3B |
|  |  | PP2AB''γ | G5PR, C14orf10 | PPP2R3C |
|  | B''' | PP2AB''' | PR53, PTPA | PTPA |
| Catalytic | C | PP2ACα | RP-C | PPP2CA |
|  |  | PP2ACβ |  | PPP2CB |

Supplementary Table 1

Human PP2A subunits were modified the article by Lubbers ER et al. in *J Mol Cell Cardiol*, 2016.
