# Supplementary material for: Sephin1 suppresses ER stress-induced cell death by inhibiting the formation of PP2A holoenzyme
Source: Cell Death Dis. 2025 Feb 19;16(1):117. doi: 10.1038/s41419-025-07450-1 (PMC11840111; doi:10.1038/s41419-025-07450-1)
Supplement: Supplementary file 3 — Original Western Blotting Images [file 41419_2025_7450_MOESM3_ESM.pdf]

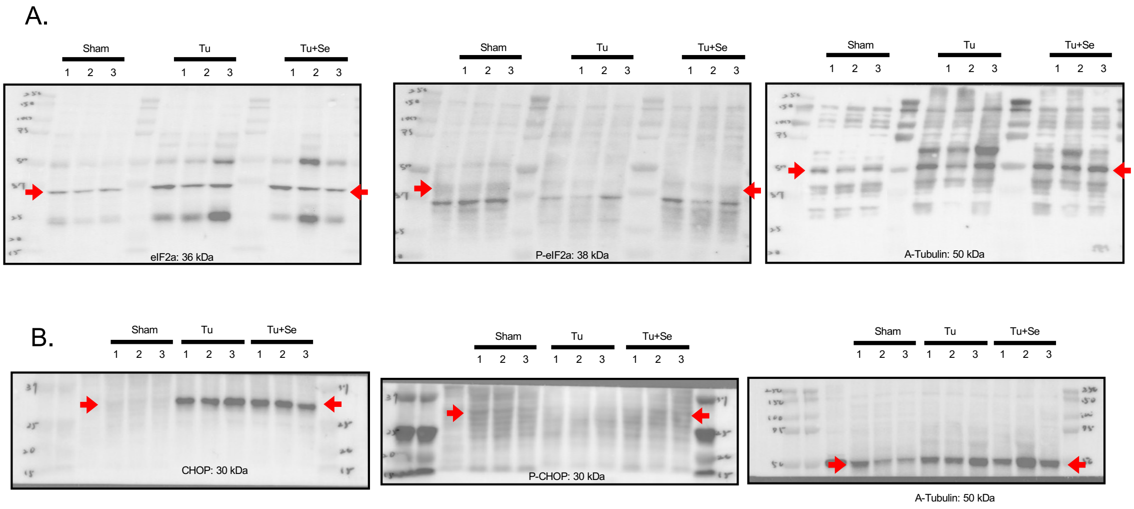

A: Western blotting images for Supplementary Figure 4

B: Western blotting images for Figure 3B&C.

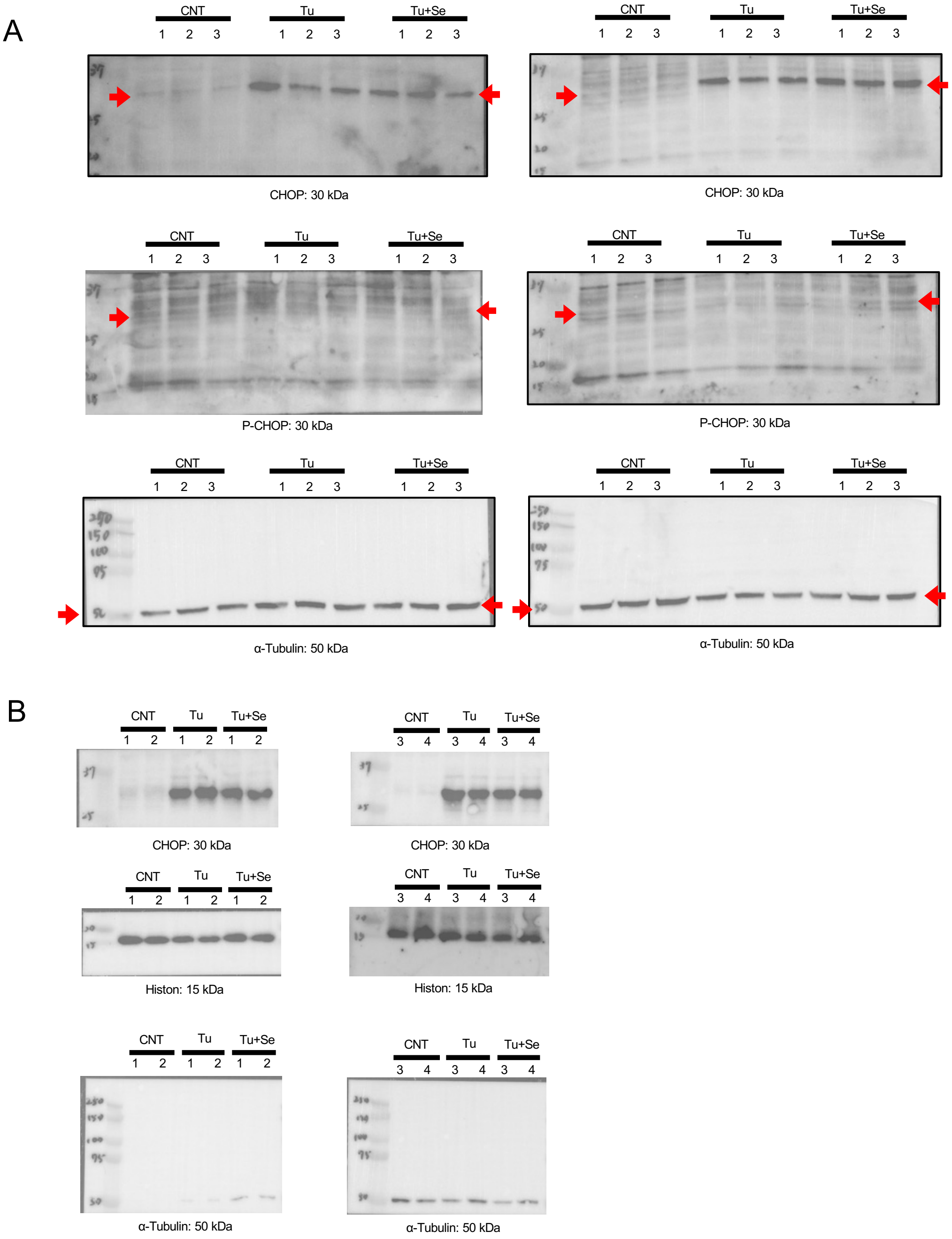

A: Western blotting whole images for Figure 3D

B: Western blotting whole images for Figure 3E

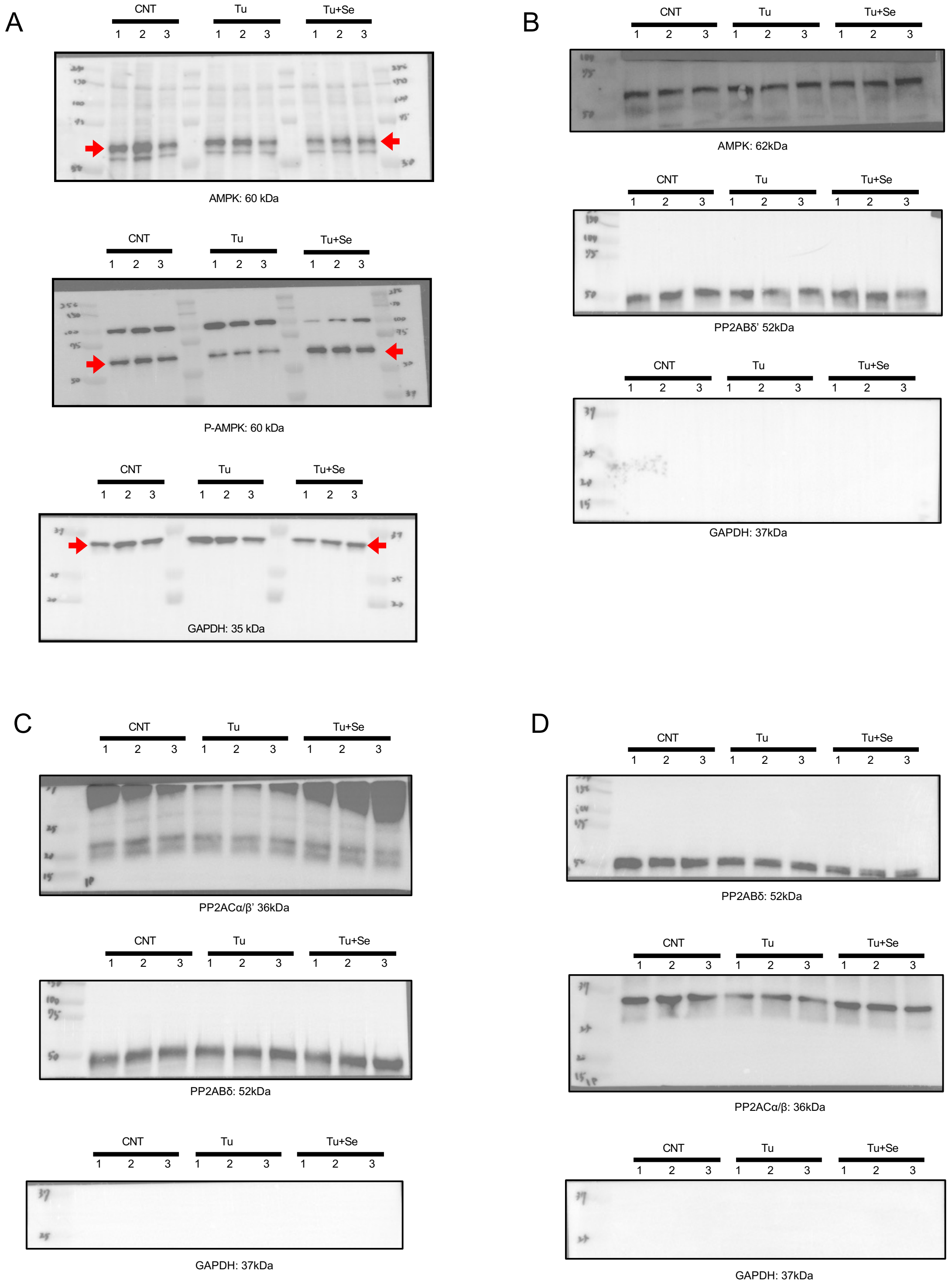

Western blotting whole images

A for Figure 5E

B for Figure 5F

C for Figure 5G

D for Figure 5H
